# Transarterial chemoembolization plus atezolizumab and bevacizumab in patients with intermediate hepatocellular carcinoma: a single-arm, phase 2 trial

**DOI:** 10.1038/s41392-025-02427-0

**Published:** 2025-10-06

**Authors:** Kang Wang, Jinkai Feng, Hongming Yu, Yuqiang Cheng, Yanjun Xiang, Zonghan Liu, Yingyi Qin, Enyu Liu, Yunfeng Shan, Chen Fan, Jian Zhai, Dandan He, Hongkun Zhou, Yufu Tang, Jie Shi, Weixing Guo, Maolin Yan, Luowen Yu, Masatoshi Kudo, Shuqun Cheng

**Affiliations:** 1 Department of Hepatic Surgery VI, Eastern Hepatobiliary Surgery Hospital, Naval Medical University, Shanghai, China; 2 Shanghai Hepatobiliary Cancer Research Center, Naval Medical University, Shanghai, China; 3 Department of Hepatic Surgery, the Oncology Center, Shanghai General Hospital, Shanghai Jiaotong University School of Medicine, Shanghai, China; 4 Department of Hepatobiliary Surgery, No. 971 Hospital of the Chinese People’s Liberation Army Navy, Qingdao, China; 5 Department of Hepatobiliary and Pancreatic Surgery, The First Affiliated Hospital of Wenzhou Medical University, Wenzhou, China; 6 Department of Health Statistics, Naval Medical University, Shanghai, China; 7 Department of Hepatobiliary Surgery, Qilu Hospital of Shandong University, Jinan, China; 8 Department of Interventional Radiology, The Affiliated Wuxi People’s Hospital of Nanjing Medical University, Wuxi, China; 9 Department II of Interventional Radiology, Eastern Hepatobiliary Surgery Hospital, Naval Medical University, Shanghai, China; 10 Department of Hepatobiliary and Pancreatic Surgery, The First Hospital of Jiaxing, Affiliated Hospital of Jiaxing University, Jiaxing, China; 11 Department of Hepatobiliary and Thyroid Surgery, General Hospital of Northern Theater Command, Shenyang, China; 12 Department of Hepatobiliary and Pancreatic Surgery, Fujian Provincial Hospital, Fuzhou, China; 13 Department of Biostatistics and Bioinformatics, Duke University School of Medicine, Durham, NC US; 14 Department of Gastroenterology and Hepatology, Kindai University, Osaka, Japan; 15 Department of Cell Biology, College of Medicine, Jiaxing University, Jiaxing, China

**Keywords:** Clinical trials, Cancer

## Abstract

Transarterial chemoembolization (TACE) is the standard treatment for intermediate-stage hepatocellular carcinoma (HCC), yet its efficacy as a standalone therapy remains suboptimal. This phase 2 trial (ChiCTR2100049829) evaluated the feasibility and safety of TACE combined with atezolizumab and bevacizumab in patients with intermediate-stage HCC. Participants received TACE followed by atezolizumab and bevacizumab until disease progression, unacceptable toxicity, or death. The primary endpoint was objective response rate (ORR) assessed per Response Evaluation Criteria in Solid Tumors (RECIST) v1.1. Secondary endpoints included progression-free survival (PFS), overall survival (OS), ORR by modified RECIST (mRECIST), disease control rate (DCR), time to response (TTR), duration of response (DOR), and adverse events (AEs). Between August 21, 2021 and April 10, 2023, 45 patients were enrolled. As of the data cutoff on September 30, 2024, the median follow-up was 26.7 months. The ORR was 47% per RECIST v1.1 and 67% per mRECIST. Median PFS was 17.9 months, and median OS was 33.0 months. The DCR was 87% (RECIST v1.1) and 91% (mRECIST). Median TTR was 11.9 weeks (RECIST v1.1) and 4.9 weeks (mRECIST), with median DOR of 36.6 weeks (RECIST v1.1) and 44.4 weeks (mRECIST). Of the 45 patients, 44 experienced AEs of any grade, with 20 reporting grade 3-4 AEs; no grade 5 AEs were observed. TACE combined with atezolizumab and bevacizumab appears safe and feasible for intermediate-stage HCC, supporting further investigation in larger studies.

## Introduction

Globally, hepatocellular carcinoma (HCC) is the sixth most commonly diagnosed cancer and the third leading cause of cancer-related mortality, representing a substantial public health burden.^1^ The burden of HCC is disproportionately high in regions endemic for chronic hepatitis B virus (HBV) infection, particularly in China, where HBV remains a major etiological driver. Despite considerable improvements in surveillance programs and diagnostic imaging techniques that facilitate early detection, approximately 80% of patients are still diagnosed at intermediate or advanced stages of the disease.^2,3^ This late presentation severely limits the applicability of curative treatments, thereby confining many patients to palliative approaches and contributing to persistently poor survival outcomes.

Intermediate-stage HCC, as classified by the Barcelona Clinic Liver Cancer (BCLC) staging system, represents a notably heterogeneous population that accounts for 20–30% of all HCC cases.^4,5^ Transarterial chemoembolization (TACE) has served as the standard of care for this patient population for more than two decades.^6^ TACE combines the delivery of high-dose chemotherapy directly into the tumor-feeding arteries with subsequent embolization of these vessels, inducing both cytotoxic effects and ischemic necrosis.^7,8^ Despite its widespread use, the efficacy of TACE is highly variable and often suboptimal in patients with multinodular involvement or large tumor burdens. Objective response rates (ORRs) remain modest, at approximately 30%, and three-year survival rates are disappointingly low, estimated at only 26%.^9–11^ Furthermore, repeated TACE sessions can lead to progressive deterioration of liver function due to ischemic insults and chemotherapy-induced toxicity, which in turn exacerbates patient prognosis and complicates further treatment.^12,13^ These limitations underscore the urgent need for more effective therapeutic strategies that can enhance and sustain the benefits of TACE.

Recent breakthroughs in systemic therapy for HCC, particularly involving immune checkpoint inhibitors and anti-angiogenic agents, have reshaped the treatment landscape and opened new avenues for combination strategies.^14^ The TACTICS trial provided initial evidence that combining TACE with the multikinase inhibitor sorafenib could significantly extend progression-free survival (PFS) compared to TACE alone.^15^ Although subsequent studies, such as TACE-2, failed to replicate these benefits, they collectively highlighted the potential of integrating locoregional and systemic treatments and spurred further clinical investigations.^16^ A paradigm shift occurred with the landmark IMbrave150 trial, which established atezolizumab (an anti-PD-L1 antibody) plus bevacizumab (an anti-vascular endothelial growth factor [VEGF] antibody) as a new first-line therapy for unresectable HCC, demonstrating significant improvements in both PFS and overall survival (OS) compared to sorafenib.^17^ The combination operates through a dual mechanism: bevacizumab inhibits VEGF-mediated angiogenesis and promotes vascular normalization, which may improve drug delivery and alleviate hypoxia, while atezolizumab reinvigorates antitumor immunity by blocking PD-L1-mediated T-cell suppression.^18–21^ Importantly, TACE-induced tumor necrosis is known to release tumor-associated antigens and pro-inflammatory signals, priming the immune system and synergizing with immunotherapy.^22^ Building on this rationale, recent trials such as EMERALD-1 and LEAP-012 have provided encouraging evidence that combining TACE with PD-L1/PD-1 inhibitors and anti-VEGF agents (either monoclonal antibodies or multikinase inhibitors) significantly improves radiological response rates and PFS compared to TACE alone.^23,24^ These findings suggest that such combinations can leverage complementary mechanisms of action to achieve enhanced antitumor activity. In particular, the combination of atezolizumab and bevacizumab may not only counteract TACE-induced hypoxia and compensatory angiogenesis but also amplify innate and adaptive immune responses through antigen release and immune checkpoint blockade.

Given this compelling scientific premise and emerging clinical evidence, we conducted a single-arm, multicenter, phase 2 trial to evaluate the preliminary efficacy and safety of combining TACE with atezolizumab and bevacizumab in patients with intermediate-stage HCC. This study aims to address the unmet need for more effective treatment regimens in this population, overcome the limitations of TACE monotherapy, and explore the therapeutic potential of multimodal treatment approaches that integrate locoregional and systemic therapies.

## Results

### Patient characteristics

**Fig. 1:**
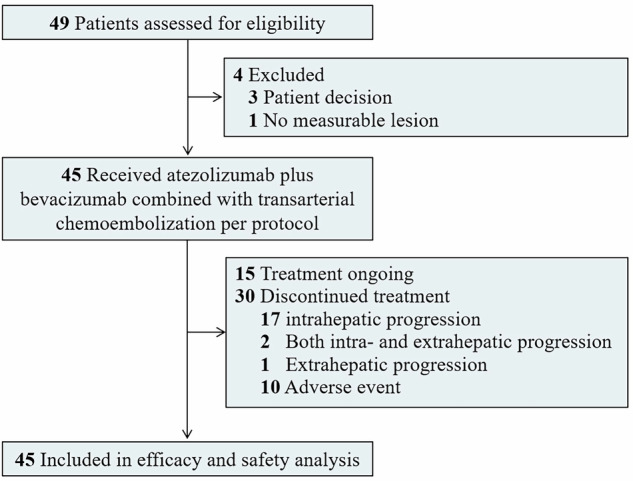
Trial profile and patient flowchart

Between August 21, 2021 and April 10, 2023, 49 patients were screened, 45 of whom met the eligibility criteria and were enrolled in the study. All 45 patients were included in both the efficacy and safety analyses (Fig. 1). Baseline demographic and clinical characteristics of the patients are summarized in Table 1. The median age of the study cohort was 55 years (range, 30–69 years), and 87% were male. HBV infection was present in 36 patients (80%), and an Eastern Cooperative Oncology Group (ECOG) performance status of 0 was also observed in 36 patients (80%). All patients had BCLC stage B disease, with 13 (29%) meeting the up-to-seven criteria and 32 (71%) exceeding them. Baseline alpha-fetoprotein (AFP) levels >400 ng/mL were reported in 16 patients (36%), and protein induced by vitamin K absence or antagonist-II (PIVKA-II) levels >400 mAU/mL in 26 patients (58%). Radiographic assessment showed a median maximum tumor diameter of 5.0 cm (interquartile range [IQR], 4.1–7.9), and a median sum of the largest tumor diameters of 10.0 cm (IQR, 8.9–12.9). Twenty-three patients (51%) had two intrahepatic tumors, while 22 (49%) had three or four lesions. Patients received a median of 17 cycles (range, 3–30) of atezolizumab-bevacizumab combination therapy and 2 TACE procedures (range, 1–4).

**Table 1:** Baseline demographic and tumor characteristics

	Patients (n = 45)
Median age, years (range)	55 (30–69)
Sex	
Male	39 (87%)
Female	6 (13%)
Etiology	
Hepatitis B	36 (80%)
Hepatitis C	4 (9%)
Alcohol abuse	2 (4%)
Cryptogenic	3 (7%)
Eastern Cooperative Oncology Group performance status	
0	36 (80%)
1	9 (20%)
Child-Pugh score	
A5	33 (73%)
A6	12 (27%)
BCLC stage B	
Within up-to-seven criteria^a^	13 (29%)
Beyond up-to-seven criteria^a^	32 (71%)
Albumin-bilirubin grade	
Grade 1	28 (62%)
Grade 2	17 (38%)
ALT, U/L	
≤40 U/L	30 (67%)
>40 U/L	15 (33%)
PT, s	
≤13 s	29 (64%)
>13 s	16 (36%)
AFP, ng/mL	
≤400 ng/mL	29 (64%)
>400 ng/mL	16 (36%)
PIVKA-II, mAU/mL	
≤400 mAU/mL	19 (42%)
>400 mAU/mL	26 (58%)
Number of intrahepatic lesions	
Two	23 (51%)
Three	15 (33%)
Four	7 (16%)
Median size of the largest lesion, cm (IQR)	5.0 (4.1–7.9)
Median sum of the largest diameters of lesions, cm (IQR)	10.0 (8.9–12.9)
Number of TACE, median (range)	2 (1–4)
Cycle of atezolizumab plus bevacizumab, median (range)	17 (3–30)

Data are n (%) unless otherwise specified

^a^Sum of the largest tumor diameter (cm) and the number of tumors greater than seven

*BCLC* Barcelona Clinic Liver Cancer, *ALT* alanine aminotrasferase, *PT* prothrombin time, *AFP* alpha-fetoprotein, *PIVKA-II* protein induced by vitamin K absence or antagonist-II, *mAU* milli-Anson unit, *IQR* interquartile range, *TACE* transarterial chemoembolization.

### Primary endpoint

**Fig. 2:**
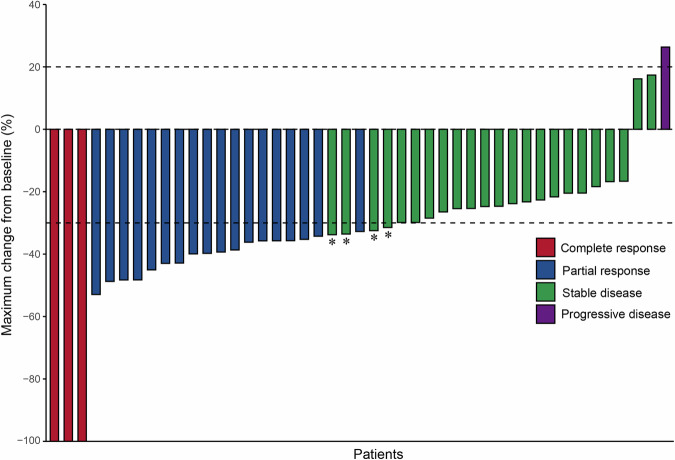
Waterfall plot showing the percentage maximum change from baseline in the sum of the longest diameter of target lesions in each of the 45 patients, according to treatment response per RECIST v1.1 criteria. RECIST, Response Evaluation Criteria in Solid Tumors. Dotted lines represent the definition of partial response and progressive disease per RECIST v1.1 criteria. * The partial response was not sustained for two consecutive assessment cycles

This study evaluated ORR by Response Evaluation Criteria in Solid Tumors (RECIST) v1.1 as the primary endpoint. The waterfall plot in Fig. 2 demonstrates the maximum percentage change from baseline in the sum of longest diameters (SLD) of target lesions, with four patients (9%) achieving a > 50% reduction in SLD. Among the 45 patients, three (7%) achieved complete response, 18 (40%) had partial response, 23 (51%) showed stable disease, and one (2%) experienced progressive disease. This results in an ORR of 47% (21/45, one-sided 90% confidence interval [CI] ≥36%) and disease control rate (DCR) of 87% (39/45, two-sided 95% CI 73–95%). The median time to response (TTR) was 11.9 weeks (IQR, 10.1–19.8) from treatment initiation. The median duration of response (DOR) reached 36.6 weeks (IQR, 14.9–46.9). Sustained antitumor activity was observed, with 47% and 29% of patients maintaining clinical benefit for at least 3 and 6 months, respectively (Table 2).

**Table 2:** Confirmed antitumor activity, assessed by RECIST v1.1

Variables	All patients per investigator review (n = 45)
Objective response rate^a^	21 (47%, ≥36%)
Complete response	3 (7%, ≥3%)
Partial response	18 (40%, ≥30%)
Stable disease	23 (51%, ≥41%)
Progressive disease	1 (2%, ≥0.2%)
Disease control rate^b^	39 (87%, 73–95)
Median time to response, weeks (IQR)	11.9 (10.1–19.8)
Median duration of response, weeks (IQR)	36.6 (14.9–46.9)
Duration of response ≥3 months	21 (47%)
Duration of response ≥6 months	13 (29%)

ORR, CR, PR, SD, and PD are described as n (%, ≥ one-sided 90% CI) of 45 patients; DCR is described as n (%, two-sided 95% CI)

*RECIST* response evaluation criteria in solid tumors

^a^Complete response or partial response for at least 4 weeks

^b^Complete response, partial response, or stable disease for at least 6 months

### Secondary endpoints

**Fig. 3:**
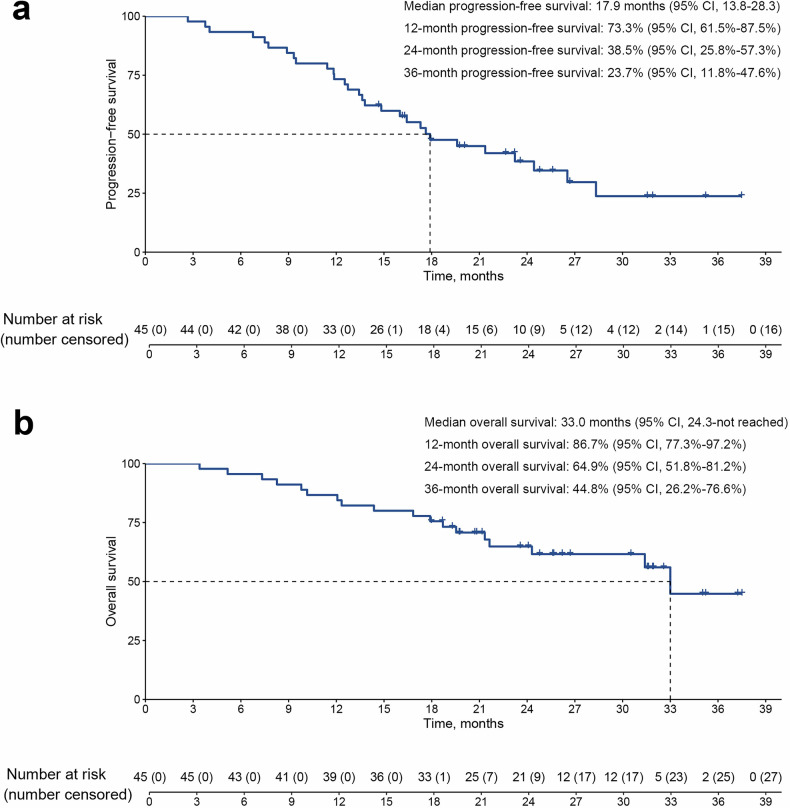
Kaplan-Meier survival curves. **a** Progression-free survival, per patient (*n* = 45); **b** Overall survival, per patient (*n* = 45)

The secondary endpoints included PFS, OS, and additional efficacy measures. As of the data cutoff on September 30, 2024, the median follow-up was 26.7 months (95% CI, 25.4-28.0). The median PFS was 17.9 months (95% CI, 13.8–28.3). The 1-, 2-, and 3-year PFS rates were 73.3% (95% CI, 61.5–87.5%), 38.5% (95% CI, 25.8–57.3%), and 23.7% (95% CI, 11.8–47.6%), respectively (Fig. 3a). The median OS was 33.0 months (95% CI, 24.3-not reached), with corresponding 1-, 2-, and 3-year OS rates of 86.7% (95% CI, 77.3–97.2%), 64.9% (95% CI, 51.8–81.2%) and 44.8% (95% CI, 26.2–76.6%), respectively (Fig. 3b). Per modified RECIST (mRECIST), 7 of 45 patients (16%) achieved complete response, 23 (51%) attained partial response, and 14 (31%) had stable disease, resulting in an ORR of 67% (30/45, one-sided 90% CI ≥ 56%) and a DCR of 91% (41/45, two-sided 95% CI 79–98%). The median TTR was 4.9 weeks (IQR, 4.3–11.2), while the median DOR was 44.4 weeks (IQR, 33.5–66.0), with 67% and 58% of patients maintaining a response for at least 3 and 6 months, respectively (Supplementary Table 1, Supplementary Figure 1).

### Safety

Forty-four patients (98%) reported adverse events (AEs) of any grade and 20 (44%) experienced grade 3/4 AEs. Ten patients (22%) who experienced grade 3/4 AEs permanently discontinued this treatment regimen. No grade 5 AEs occurred (Table 3). Among these, the most frequently occurring grade 1–2 AEs were fatigue (21/45, 47%), nausea (18/45, 40%), pyrexia (15/45, 33%), decreased appetite (13/45, 29%), increased alkaline phosphatase (13/45, 29%), and hyponatraemia (13/45, 29%). The most commonly observed grade ≥3 AEs were elevated aspartate aminotransferase (7/45, 16%), increased alanine aminotransferase (5/45, 11%), hypertension (3/45, 7%), proteinuria (3/45, 7%), hypercreatinine (3/45, 7%), diarrhea (2/45, 4%), increased bilirubin (2/45, 4%), and immune-related hepatitis (2/45, 4%).

**Table 3:** Adverse events for all 45 patients

	Grade 1–2	Grade 3	Grade 4	Grade 5
**Any adverse events**	24 (53%)	16 (36%)	4 (9%)	0
**Constitutional**				
Fatigue	21 (47%)	1 (2%)	0	0
Pyrexia	15 (33%)	1 (2%)	0	0
Weight loss	9 (20%)	0	0	0
Infusion-related reaction	5 (11%)	0	0	0
Headache or dizziness	6 (13%)	1 (2%)	0	0
Hypertension	9 (20%)	3 (7%)	0	0
**Skin**				
Rash	8 (18%)	1 (2%)	0	0
Pruritus	6 (13%)	0	0	0
Mucositis oral	5 (11%)	0	0	0
**Respiratory**				
Cough	3 (7%)	0	0	0
Chest discomfort	1 (2%)	0	0	0
**Gastrointestinal**				
Decreased appetite	13 (29%)	0	0	0
Nausea	18 (40%)	0	0	0
Vomiting	10 (22%)	0	0	0
Diarrhea	5 (11%)	1 (2%)	1 (2%)	0
Constipation	2 (4%)	0	0	0
Gastrointestinal bleeding	1 (2%)	0	0	0
**Hepatic**				
Increased alanine aminotransferase	9 (20%)	4 (9%)	1 (2%)	0
Increased aspartate aminotransferase	11 (24%)	5 (11%)	2 (4%)	0
Increased bilirubin	10 (22%)	2 (4%)	0	0
Increased gamma-glutamyl transferase	3 (7%)	1 (2%)	0	0
Increased alkaline phosphatase	13 (29%)	1 (2%)	0	0
Decreased albumin	7 (16%)	0	0	0
**Laboratory**				
Proteinuria	8 (18%)	3 (7%)	0	0
Anemia	8 (18%)	0	0	0
Thrombocytopenia	9 (20%)	0	1 (2%)	0
Neutropenia	2 (4%)	0	0	0
Leukopenia	5 (11%)	0	0	0
Hyponatraemia	13 (29%)	0	0	0
Hypokalaemia	5 (11%)	0	1 (2%)	0
Hypercreatinine	4 (9%)	3 (7%)	0	0
Hypothyroidism	6 (13%)	0	0	0
**Immune-related**				
Hepatitis	3 (7%)	2 (4%)	0	0
Pneumotitis	1 (2%)	0	0	0

### Discussion

To our knowledge, this phase 2 trial represents one of the earliest prospective studies of TACE combined with atezolizumab and bevacizumab for intermediate HCC, demonstrating encouraging clinical efficacy with a 47% ORR per RECIST v1.1 and median PFS of 17.9 months. These results are comparable to or exceed those reported in related studies, such as the IMbrave150 and EMERALD-1 trials, which further support the efficacy of this triple therapy.^17,23^

The therapeutic rationale for this combination lies in its synergistic mechanisms. TACE mediates extensive tumor necrosis, thereby releasing tumor-associated antigens and initiating an immune response. Atezolizumab enhances anti-tumor immunity by blocking PD-L1 and promoting T-cell activity.^25–27^ However, the hypoxic environment following TACE stimulates VEGF production, promoting angiogenesis and immunosuppression.^28,29^ Bevacizumab, a humanized monoclonal antibody, potentially inhibits post-TACE angiogenesis through VEGF blockade.^30^ Bevacizumab counters this by inhibiting VEGF, normalizing tumor vasculature, and reprogramming the immune microenvironment.^31,32^ Together, combination treatment with these agents addresses the limitations of TACE monotherapy, offering a comprehensive therapeutic strategy to intermediate HCC.

The CHANCE001 study reported significant improvements in PFS (P = 0.015), OS (P = 0.037), and ORR (P < 0.001) with TACE plus PD-(L)1 inhibitors and molecular targeted therapies compared to TACE monotherapy in Chinese patients with predominantly advanced HCC.^33^ Recent trials, including EMERALD-1 and LEAP-012, have significantly advanced the clinical application of the TACE plus immunotherapy and anti-angiogenesis triplet regimen. In the LEAP-012 trial, TACE combined with lenvatinib and pembrolizumab significantly prolonged PFS compared to TACE alone (P = 0.0002) in patients with unresectable, non-metastatic HCC, particularly for patients in BCLC stage B.^24^ However, the trial reported a high incidence of grade 3–4 AEs (70%, 165/237), likely due to the broad inhibitory effects of lenvatinib, a multi-target tyrosine kinase inhibitor, which may increase treatment-related toxicity. In contrast, the safety profile of bevacizumab appears more controllable. The EMERALD-1 trial evaluated TACE in combination with durvalumab (an anti-PD-L1 monoclonal antibody) and bevacizumab in patients with unresectable HCC amenable to embolization.^23^ In the present study, we observed numerically higher outcomes compared to the EMERALD-1 trial, with an ORR of 47% vs. 44% and a median PFS of 17.9 vs. 15.0 months.^23^ This improvement could be attributed in part to the efficacy of the atezolizumab-bevacizumab combination, which has shown a synergistic effect in advanced HCC,^17^ potentially being further enhanced when integrated with TACE. Additionally, the exclusion of BCLC stage C patients (0% in this study vs. ~17% in the EMERALD-1 trial) may have enabled more precise targeting of potentially benefiting populations. The phase III TALENTACE study (NCT047126430), assessing atezolizumab, bevacizumab, and on-demand TACE in patients with unresectable HCC who have not received prior systemic treatment, demonstrated a statistically significant and clinically meaningful improvement in TACE-PFS (time from randomization to untreatable progression, TACE failure/refractoriness, or death by any cause).^34,35^

These trial results also underscore areas that require further research. Determining the optimal sequence and timing of TACE and systemic therapy is crucial. For example, in the LEAP-012 trial, pembrolizumab and lenvatinib were typically started 2 to 4 weeks before the first TACE treatment, whereas in this study and the EMERALD-1 trial, systemic treatment began after the first TACE. Both approaches are reasonable: initiating systemic therapy before TACE may heighten the efficacy of TACE by normalizing the tumor abnormal vessel, decreasing intratumoral interstitial density and increasing drug delivery;^22,36^ while performing TACE prior to systemic therapy may potentiate tumor antigen release, increase CD8 T-cell infiltration and upregulate PD-L1 expression.^37,38^ Furthermore, it is worth exploring whether this combination therapy is applicable to patients beyond BCLC stage B.

Regarding safety, no new or unexpected AEs were observed. Among the 45 patients, 24 (53%) patients encountered grade 1–2 AEs, 16 (36%) patients experienced grade 3 AEs, 4 (9%) patients developed grade 4 AEs, and no grade 5 AEs occurred. Most grade 1–2 AEs were resolved with supportive care, and treatment adjustments were not required. For grade 3/4 AEs, such as immune-related hepatitis and elevated transaminases, treatment was temporarily interrupted, and corticosteroids or other immunosuppressive agents were administered. 10 (22%) patients permanently discontinued treatment due to AEs, and there were no deaths related to AEs, indicating that the therapy is generally well-tolerated and manageable.

Despite these promising findings, several limitations must be acknowledged. While the observed ORR and PFS suggest promising antitumor activity of TACE combined with atezolizumab and bevacizumab, the single-arm design limits definitive conclusions regarding the regimen’s efficacy. Without a randomized control group, the observed outcomes cannot be attributed solely to the treatment. Comparisons to historical controls indicate that the results are consistent with or exceed those of similar therapeutic strategies, yet these comparisons should be interpreted cautiously. Larger phase 3 randomized controlled trials are necessary to validate these findings. Additional limitations include the relatively small sample size and the predominance of patients with HBV-related HCC. Previous studies indicate that HBV-related HCC may respond favorably to immunotherapy than non-viral HCC.^39,40^ This characteristic may restrict the generalizability of these results to broader HCC patients with diverse etiologies. As an exploratory phase 2 study, the primary objective was to assess feasibility and generate hypotheses rather than provide definitive evidence.

Overall, this trial suggests that TACE combined with atezolizumab and bevacizumab is a feasible and well-tolerated treatment for intermediate-stage HCC. The encouraging efficacy and favorable safety profile warrant further validation in larger randomized controlled trials.

## Methods

### Study design and patients

This study was an investigator-initiated, single-arm, multicenter phase 2 trial, which investigated the efficacy and safety of TACE combined with atezolizumab plus bevacizumab in patients with intermediate-stage HCC. The study was conducted at the Eastern Hepatobiliary Surgery Hospital, Qilu Hospital of Shandong University, the First Affiliated Hospital of Wenzhou Medical University, the Affiliated Wuxi People’s Hospital of Nanjing Medical University, The First Hospital of Jiaxing, and Fujian Provincial Hospital. A study management committee comprising principal investigators from all participating centers was established to ensure protocol adherence. All patient eligibility and outcomes were centrally reviewed and confirmed by this committee. To ensure data consistency and integrity across study sites, an independent centralized team implemented standardized follow-up procedures, maintaining comprehensive records of treatment administration, radiographic responses, therapeutic outcomes, and adverse events.

The inclusion criteria for this study were as follows: 1) aged between 18 and 70 years; 2) radiologically or histologically diagnosed HCC; 3) BCLC stage B; 4) at least one measurable lesion per RECIST v1.1; 5) ECOG performance status 0–1; 6) preserved liver function (Child-Pugh class A); 7) adequate bone marrow, hepatic and renal functions.

Key exclusion criteria comprised: 1) prior locoregional or systemic treatment for HCC; 2) documented hepatic encephalopathy; 3) untreated or suboptimally managed varices with active bleeding or high risk of bleeding under endoscopy; 4) clinically significant gastrointestinal hemorrhage requiring intervention within 4 weeks preceding enrolment; 5) concurrent autoimmune disorders requiring immunosuppression, primary or acquired immunodeficiency states, history of solid organ or hematopoietic stem cell transplantation, or other coexisting malignancies; 6) undergoing major surgical procedure within 4 weeks; 7) immunosuppressive or systemic steroid use within 2 weeks; 8) decompensated vital organ failure.

### Ethics

This study adhered to the International Conference on Harmonization Good Clinical Practice guidelines, the ethical principles of the Declaration of Helsinki, and relevant local regulatory requirements. The Institutional Review Board of Eastern Hepatobiliary Surgery Hospital approved the study protocol (approval number: EHBHKY2021-K-015) prior to patient enrollment. Written informed consent was obtained from all participants, and the trial was prospectively registered with the Chinese Clinical Trial Registry (registration number: ChiCTR2100049829).

### Procedures

Baseline imaging examination was conducted within 4 weeks prior to enrolment. Standard TACE was administered within the protocol-specified 3-day window following enrollment, performed under local anesthesia with super selective delivery of chemotherapeutic agents to tumor-specific arterial branches. Then an emulsion of lipiodol and pirarubicin was administered through the microcatheter into the tumor-feeding arteries, followed by embolization using absorbable gelatin sponge particles until complete flow stagnation was documented angiographically. TACE repetition was considered if follow-up imaging revealed residual viable lesions (evidenced by arterial-phase enhancement on contrast-enhanced computed tomography [CT] or magnetic resonance imaging [MRI]) and the patient’s liver function remained adequate (Child-Pugh A5 or A6). If contrast-enhanced imaging showed adequate iodine oil accumulation, substantial tumor necrosis, and no evidence of disease progression or new lesions, a second session of TACE was not performed. TACE could be repeated for small new intrahepatic lesions considered treatable without evidence of systemic progression. TACE treatment discontinuation occurred in cases of confirmed disease progression, persistent liver dysfunction (Child-Pugh score exceeding A6), or any condition rendering TACE infeasible, including intolerable toxicity or patient refusal.

Systemic therapy would be initiated 2–14 days following the first TACE procedure, upon recovery of liver function. The treatment regimen comprised intravenous atezolizumab (1200 mg fixed dose) combined with weight-adjusted bevacizumab (15 mg/kg), administered every 21 days. Treatment was maintained until the occurrence of any of the following: disease progression, unacceptable toxicity, patient withdrawal of consent, or investigator-determined discontinuation. Dose modification of systemic drugs was not permitted; however, permanent discontinuation of individual study drugs was permitted based on clinical necessity.

Standard oral nucleos(t)ide analogues (entecavir 0.5 mg or tenofovir disoproxil 300 mg daily) were prescribed for all enrolled patients with detectable HBV viremia. Patients exhibiting HBV-DNA levels >2000 IU/mL received a minimum of one week of antiviral therapy prior to initiating combination treatment. Baseline assessment consisted of a detailed medical record review, physical examination, laboratory analyses (complete blood count, coagulation profile, hepatic and renal function tests, serum AFP and PIVKA-II levels), and radiographic imaging (chest CT, and contrast-enhanced abdominal CT and/or MRI). On-treatment follow-up was conducted at 3-week intervals and involved clinical evaluation, laboratory monitoring, and documentation of adverse events. Imaging examinations (enhanced CT or MRI) were conducted every 6 weeks (±7 days) after treatment initiation until disease progression or unacceptable toxicities. Tumor response was classified as complete response, partial response, stable disease, or progressive disease based on both RECIST v1.1 and mRECIST criteria.^41,42^ Two independent radiologists, each with more than 15 years of diagnostic experience, performed all radiographic assessments. In cases of diagnostic uncertainty or disagreement between radiologists, a third independent blinded radiologist with specialized expertise conducted adjudication to determine the final assessment. Following treatment discontinuation, all participants entered long-term follow-up with survival status monitored quarterly during the first year and biannually thereafter.

### Outcomes

The primary endpoint was ORR assessed according to RECIST v1.1 criteria. Secondary endpoints included PFS, OS, ORR by mRECIST criteria, DCR, TTR, DOR, and AEs. ORR was calculated as the proportion of patients achieving a confirmed complete response (CR) or partial response (PR) lasting at least 4 weeks. PFS was defined as the time from treatment start to the first documented radiological progression (per RECIST v1.1), new lesion appearance, or death; while OS was measured from the initiation of treatment until death from any cause. DCR represented the percentage of patients with CR, PR, or stable disease (SD) maintained for ≥6 months. TTR referred to the interval between treatment commencement and the first observed PR or CR; whereas DOR encompassed the time from initial response (CR/PR) until disease progression or death. All AEs were documented and graded according to the National Cancer Institute Common Terminology Criteria for Adverse Events version 5.0 (NCI-CTCAE v5.0).

### Statistical analysis

According to a previous study, the ORR of bevacizumab combined with TACE is 35% per RECIST v1.1 in unresectable HCC.^43^ During study design, we hypothesized that the novel combination of TACE with atezolizumab and bevacizumab could potentially elevate the ORR from 35% to 55%, though no formal statistical comparison was planned. Based on this assumption, a sample size of 44 patients was calculated to provide 90% statistical power at a one-sided α level of 0.10. To account for potential participant attrition, the target enrollment was increased to 49 patients, anticipating an approximate 10% drop-out rate. For outcome assessments, two analysis populations were predefined: the full analysis set for efficacy evaluation and the safety analysis set for safety monitoring, both including all eligible patients who received at least one cycle of systemic therapy (atezolizumab plus bevacizumab) and one TACE procedure.

Continuous variables were expressed as medians with interquartile ranges or ranges, while categorical variables were reported as frequencies with percentages. The Clopper-Pearson exact method was employed for analyses of tumor response outcomes, generating point estimates with one-sided 90% CIs for ORR, CR, PR, SD and PD, and two-sided 95% CIs for DCR. Survival outcomes, including OS and PFS, were analyzed through Kaplan-Meier estimation with corresponding 95% confidence intervals derived using the Brookmeyer-Crowley approach. All statistical analyses were conducted with R software (version 4.0.2; R Foundation for Statistical Computing). The complete study protocol is available in the Supplementary Materials.

## Supplementary information

Supplementary Figure and Table

Research Program

## Data Availability

The datasets supporting the findings of the current study are included in the manuscript and its Supplemental Materials. The datasets are available from the corresponding author upon reasonable request.
