# Supplementary material for: Transarterial chemoembolization plus atezolizumab and bevacizumab in patients with intermediate hepatocellular carcinoma: a single-arm, phase 2 trial
Source: Signal Transduct Target Ther. 2025 Oct 6;10:328. doi: 10.1038/s41392-025-02427-0 (PMC12497873; doi:10.1038/s41392-025-02427-0)
Supplement: Supplementary file 1 — Supplementary Figure and Table [file 41392_2025_2427_MOESM1_ESM.docx]

Supplementary Materials for

Transarterial chemoembolization plus Atezolizumab and Bevacizumab in Patients with Intermediate Hepatocellular Carcinoma: A Single-Arm, Phase 2 Trial

Kang Wang, Jin-Kai Feng, Hong-Ming Yu, Yu-Qiang Cheng, Yan-Jun Xiang, Zong-Han Liu, Ying-Yi Qin, En-Yu Liu, Yun-Feng Shan, Chen Fan, Jian Zhai, Dan-Dan He, Hong-Kun Zhou, Yu-Fu Tang, Jie Shi, Wei-Xing Guo, Mao-Lin Yan, Luo-Wen Yu, Masatoshi Kudo, Shu-Qun Cheng

Correspondence to: chengshuqun@aliyun.com

**This PDF file includes:**

Figure S1

Table S1


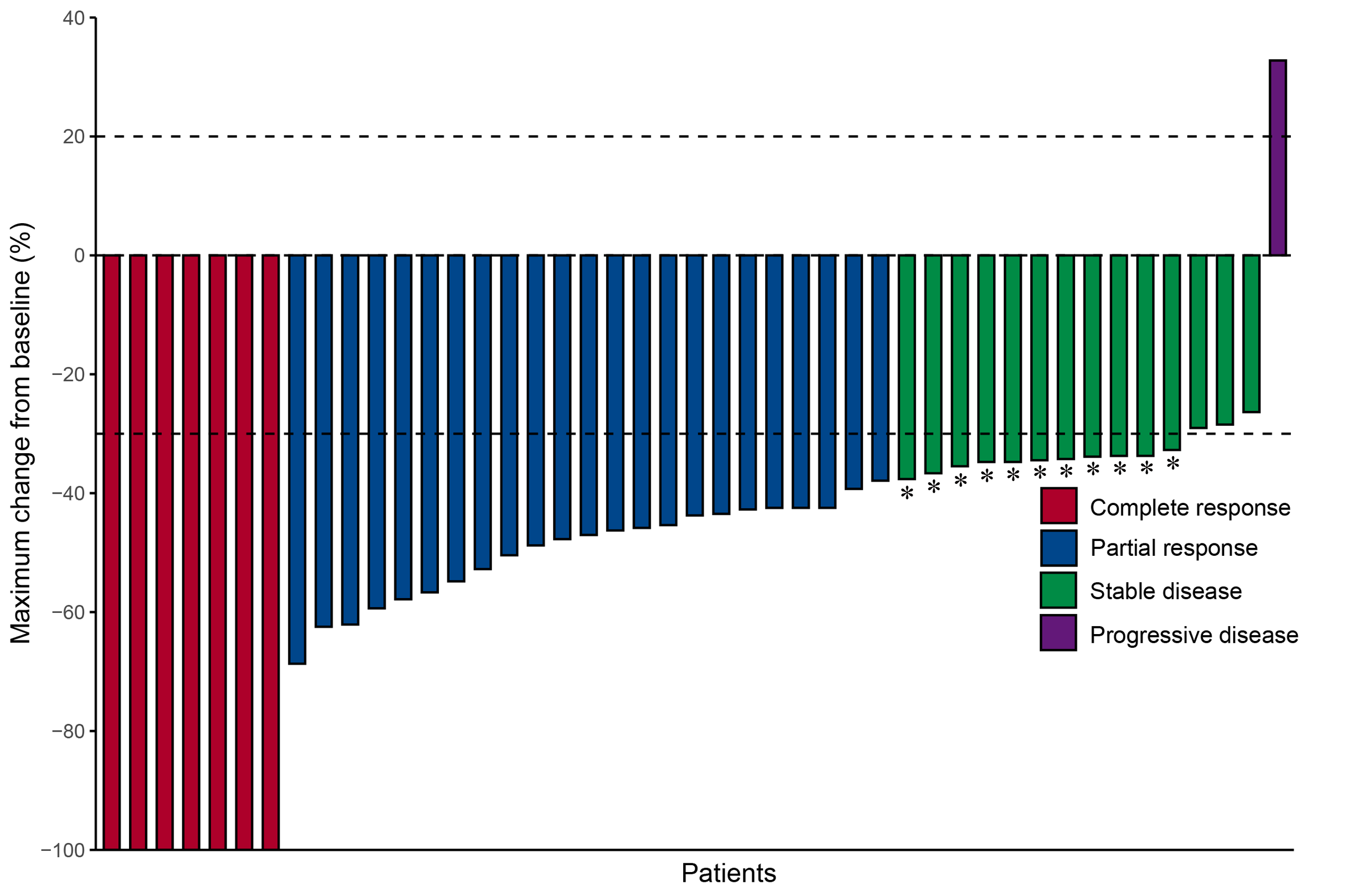


**Figure. S1:** Waterfall plot showing the percentage maximum change from baseline in the sum of the longest diameter of target lesions in each of the 45 patients, according to treatment response per mRECIST criteria. mRECIST, modified Response Evaluation Criteria in Solid Tumors. Dotted lines represent the definition of partial response and progressive disease per mRECIST criteria.

* The partial response was not sustained for two consecutive assessment cycles.

**Table S1.** Confirmed antitumor activity, assessed by modified RECIST

| Variables | All patients per investigator review (n=45) |
| --- | --- |
| Objective response rate* | 30 (67%, ≥56%)  7 (16%, ≥9%)  23 (51%, ≥41%)  14 (31%, ≥22%)  1 (2%, ≥0.2%)  41 (91%, 79–98)  4.9 (4.3–11.2)  44.4 (33.5–66.0)  30 (67%)  26 (58%) |
| Complete response |  |
| Partial response |  |
| Stable disease |  |
| Progressive disease |  |
| Disease control rate† |  |
| Median time to response, weeks (IQR) |  |
| Median duration of response, weeks (IQR) |  |
| Duration of response ≥ 3 months |  |
| Duration of response ≥ 6 months |  |

ORR, CR, PR, SD, and PD are described as n (%, ≥ one-sided 90% CI) of 45 patients; DCR is described as n (%, two-sided 95% CI).

mRECIST, modified Response Evaluation Criteria in Solid Tumors.

*Complete response or partial response for at least 4 weeks.

†Complete response, partial response, or stable disease for at least 6 months.
