# Supplementary material for: Transarterial chemoembolization plus atezolizumab and bevacizumab in patients with intermediate hepatocellular carcinoma: a single-arm, phase 2 trial
Source: Signal Transduct Target Ther. 2025 Oct 6;10:328. doi: 10.1038/s41392-025-02427-0 (PMC12497873; doi:10.1038/s41392-025-02427-0)
Supplement: Supplementary file 2 — Research Program [file 41392_2025_2427_MOESM2_ESM.docx]

**A Single-arm, Phase II Clinical Trial of TACE in**

**Combination with Atezolizumab and Bevacizumab for Intermediate-Stage Hepatocellular Carcinoma**

**Research Program**

**Study number:** ChiCTR2100049829

**Version No.:** v1.0

**Version date:** August 2021

**Clinical Research Leader and Affiliations:** Shu-Qun Cheng,

The Eastern Hepatobiliary Surgery Hospital

**Program signature page**

I have read the protocol for this study as a participating physician/statistical analyst.

I have fully discussed the purpose of this study and the content of this program

with the study leader.

I agree to conduct the study per this protocol and to comply with its requirements,

to abide by the code of ethics, and to conduct this clinical study under the guidance of

the Good Clinical Practice (GCP) for Drug Clinical Trials.

I agree that the contents of this protocol are confidential and will not be disclosed

to third parties and that the protocol’s contents will be used solely to conduct this study.

I understand that if this study was decided to terminate or suspend early at any

time for whatever reason, I will be notified in writing. Similarly, if I decide to withdraw from performing this study, I will immediately notify the study team leader unit and the principal investigator in writing.

Name: ________________

Signature: ________________

Date: ________________

**CATALOG**

**[PROGRAM SUMMARY ............................................................................................3](#ProgramSummary)**

**[ABBREVIATIONS AND DEFINITIONS ...............................................................24](#Abbreviations)**

1. **[BACKGROUND AND RATIONALE OF THE STUDY ..................................28](#BackgroundandRationaleoftheStudy)**
   1. [BACKGROUND .................................................................................................28](#Background)
   2. [RATIONALE OF THE STUDY ............................................................................33](#RationaleoftheStudy)
2. **[STUDY AIMS AND STUDY ENDPOINTS .......................................................35](#StudyAimsandStudyEndpoints)**
   1. [PRIMARY RESEARCH OBJECTIVES ................................................................35](#PrimaryResearchObjectives)
   2. [SECONDARY RESEARCH OBJECTIVES ...........................................................35](#SecondaryResearchObjectives)
   3. [PRIMARY ENDPOINTS .....................................................................................35](#PrimaryEndpoints)
   4. [SECONDARY ENDPOINTS ................................................................................36](#SecondaryEndpoints)
3. **[STUDY DESIGN ..................................................................................................37](#StudyDesign)**
   1. [GENERAL DESIGN ...........................................................................................37](#GeneralDesign)
   2. [STUDY POPULATION .......................................................................................37](#StudyPopulation)
   3. [DRUG DELIVERY PROGRAMS .........................................................................44](#DrugDeliveryPrograms)
   4. [DURATION OF THE STUDY ..............................................................................46](#DurationoftheStudy)
   5. [DISPENSING, STORAGE, ADMINISTRATION AND RECALL OF](#DispensingStorageAdministrationandRecall)

[MEDICINES ......................................................................................................46](#DispensingStorageAdministrationandRecall)

1. **[RESEARCH PROCESS ......................................................................................46](#ResearchProcess)**
   1. [SCREENING PERIOD ........................................................................................46](#ScreeningPeriod)
   2. [TREATMENT PERIOD .......................................................................................49](#TreatmentPeriod)
   3. [FOLLOW-UP PERIOD ........................................................................................5](#FollowupPeriod)0
2. **[ADVERSE EVENTS REPORTING ...................................................................50](#AdverseEventsReporting)**
   1. [ADVERSE EVENTS ...........................................................................................50](#AdverseEvents)
   2. [SERIOUS ADVERSE EVENTS ...........................................................................52](#SeriousAdverseEvents)
   3. [PATHWAYS OF ADVERSE EVENTS RECORDING AND REPORTING ................54](#PathwaysofAdverseEventsReporting)
   4. [RISK PREVENTION AND TREATMENT ............................................................56](#RiskPreventionandTreatment)
3. **[DATA COLLECTION PLAN AND STATISTICAL ANALYSIS ....................56](#DataCollectionPlanandStatisticalAnalysis)**
   1. [DATA COLLECTION PLAN ...............................................................................56](#DataCollectionPlan)
   2. [STATISTICAL ANALYSIS OF DATA SETS .........................................................56](#StatisticalAnalysisofDataSets)
   3. [STATISTICAL ANALYSIS PLAN ........................................................................56](#StatisticalAnalysisPlan)
   4. [BASIC PATIENT CHARACTERISTICS ...............................................................57](#BasicPatientCharacteristics)
   5. [EFFICACY ANALYSIS .......................................................................................57](#EfficacyAnalysis)
   6. [SAFETY ANALYSIS ..........................................................................................57](#SafetyAnalysis)
4. **[DATA MANAGEMENT ......................................................................................57](#DataManagement)**
5. **[QUALITY CONTROL AND QUALITY ASSURANCE ..................................58](#QualityControlandQualityAssurance)**
6. **[ETHICAL, REGULATORY AND ADMINISTRATIVE PRINCIPLES ........58](#EthicalRegulatoryandAdministrativePrinci)**
   1. [LOCAL LEGISLATION AND DECLARATION OF HELSINKI ..............................58](#LocalLegislationandDeclarationofHelsinki)
   2. [INFORMED CONSENT ......................................................................................59](#InformedConsent)
   3. [INDEPENDENT ETHICS COMMITTEE AND STATUTORY AUDIT](#IndependentEthicsCommitteeandStatutory)

[COMMITTEE ....................................................................................................59](#IndependentEthicsCommitteeandStatutory)

- 1. [CONFIDENTIALITY AGREEMENTS .................................................................](#ConfidentialityAgreements).60
  2. [RECORD KEEPING ...........................................................................................60](#RecordKeeping)
  3. [EARLY DISCONTINUATION OF RESEARCH ....................................................60](#EarlyDiscontinuationofResearch)

**[REFERENCES ..........................................................................................................61](#References)**

**Program Summary**

| **Study Title** | A single-arm, phase II clinical trial of TACE in combination with atezolizumab and bevacizumab for intermediate-stage hepatocellular carcinoma |
| --- | --- |
| **Study Number** | ChiCTR2100049829 |
| **Version Number** | Version 1.0 |
| **Research Purpose** | To evaluate the efficacy and safety of TACE combined with atezolizumab plus bevacizumab for patients with intermediate-stage hepatocellular carcinoma |
| **Nature of Research** | Investigator initiated, single-arm, multicenter, prospective clinical study |
| **Object of Research** | Patients with intermediate-stage (BCLC stage B) hepatocellular carcinoma |
| **Determination of Sample size** | According to a previous study, the ORR of bevacizumab combined with TACE is 35% (H_0_ = 35%) per RECIST v1.1 in unresectable HCC. Although we do not conduct any formal statistical test, at study design we expected that the new therapeutic regimen of TACE combined with atezolizumab plus bevacizumab could increase the ORR from 35% to 55% (H_1_ = 55%). A sample size of 44 patients should be enrolled to provide at least 90% power at a one-sided α level of 10%. Considering an approximately drop-out rate of 10%, a total sample size of 49 patients was planned for this study. |
| **Principal Investigator** | Shu-Qun Cheng |
| **Applicant** | The Eastern Hepatobiliary Surgery Hospital of Naval Medical University |
| **Research Design** | This is single-arm, phase 2 clinical trial in which eligible patients will receive the triple therapy consisted of TACE in combination of atezolizumab and bevacizumab.  This study is comprised of a screening period (conducted within 28 days before the first treatment initiation), a treatment period (patients were treated with the study treatment regimen), and a follow-up period (including both a safety follow-up and a survival follow-up).  **Screening period:**  Patients will be required to receive a screening assessment within 28 days before their first dose of treatment to judge and determine their eligibility for study enrolment. Patients baseline characteristics, such as age, sex, laboratory tests, radiological examinations and prior treatments, will be thoroughly collected during the screening period, and who are eligible for study enrolment will undergo TACE in combination with atezolizumab plus bevacizumab.  **Treatment period:**  Eligible patients will receive conventional TACE within 3 days of enrolment. Conventional TACE was performed under local anesthesia with selective hepatic angiography to identify the tumor-feeding vessels. Then an emulsion of lipiodol and pirarubicin was injected through the microcatheter into the tumor-feeding arteries, followed by embolization with gelatin sponge particles until complete arterial flow stasis was observed. If contrast-enhanced CT or MRI showed dense iodine oil  deposition, obvious necrosis of the lesion, and no enlargement or new tumors, a second session of TACE was not considered. The frequency of TACE was determined by the follow-up imaging results during the treatment period. If the follow-up imaging results showed residual active lesions in the liver and the patient’s liver function is adequate, TACE would be repeated. Nevertheless, TACE should be discontinued if the patient’s disease progressed or if there was a condition that made TACE infeasible, or persistent liver dysfunction occurred.  Systemic treatment will be initiated 2–14 days after the first session of TACE when liver function of patients recovered to normal. Atezolizumab plus bevacizumab is administered in cycles every 21 days. The treatment cycle is counted from the day of commencement of the agents. On the first day of each cycle, atezolizumab will be administered intravenously at a fixed dose of 1200 mg (initial intravenous injection for 60 min, and subsequent intravenous injections for 30 min if well-tolerated). Bevacizumab will be intravenously infused at a dose of 15 mg/kg at least 5 min apart (initial intravenous injection was for 90 min, then 60 min and then 30 min in turn if well-tolerated). Systemic treatment would continue until progressive disease, intolerable toxicity, refusal of treatment, and other conditions that the investigators considered necessary to discontinue treatment. Dose modifications of atezolizumab and bevacizumab will not be allowed, while permanent discontinuation of one study drug was allowed if clinically indicated.  During treatment period, patients completed different examinations on day 1 of each systemic treatment cycle every 3 weeks, including physical examination, vital signs, Eastern Cooperative Oncology Group performance status (ECOG-PS) score, and laboratory tests. The safety and adverse events are also monitored every 3 weeks during treatment period. Participants also need to complete tumor imaging evaluation (CT or MRI, evaluation method is consistent before and after) at a frequency of one time every 6 weeks (±7 days) after the first administration of the drugs. See the study flow chart for details of the specific examinations.  **Follow-up period:**  When a participant terminates treatment or withdraws from the  study, it is recommended that another thorough and comprehensive examination be performed, including physical examination, vital signs, ECOG-PS score, laboratory tests, and tumor radiological examinations (CT or MRI).  Participants will be followed up for safety evaluation for 90 days after their last cycle of systemic treatment for adverse events, concomitant medications, treatment compliance, dispensing and recovery of drugs.  Participants who discontinue treatment for reasons other than  disease progression or unacceptable toxicities will continue to be evaluated for tumor response as originally planned, with the frequency of evaluations as previously planned (1 evaluation every 6 weeks) until disease progresses, lost to follow-up, or death.  Survival follow-up is needed for all participants after termination  of treatment, every 3 months in the first year and every 6 months  thereafter, and can be done by telephone to collect survival status until  death, loss to follow-up, or other causes, etc.  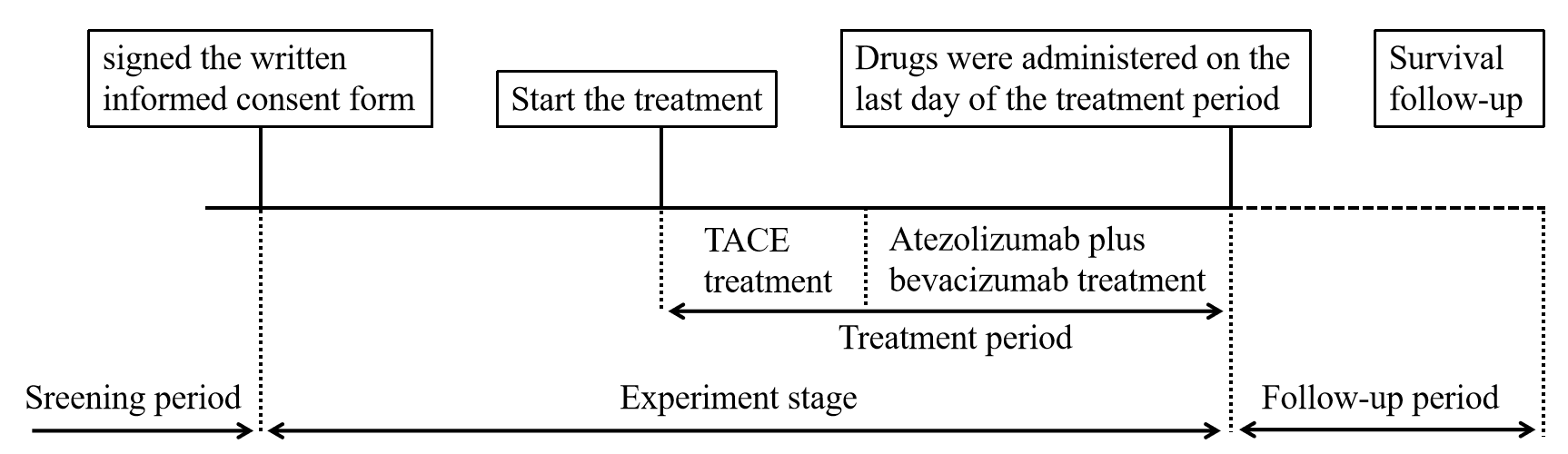 |
| **Participant Screening Criteria** | **Clinical diagnostic criteria for HCC:**   1. For patients who have hepatitis B or hepatitis C, or have cirrhosis caused by any reason, ultrasound and alpha-fetoprotein (AFP) screening should be performed at least once every 6 months. If intrahepatic nodules with a diameter of ≤2 cm are found, and at least two out of the four imaging examinations (dynamicenhanced MRI, dynamic-enhanced CT, ultrasonography, and pramiracetam dynamic-enhanced MRI) show the typical features of “fast in, fast out”, the clinical diagnosis of HCC can be made. For nodules with a diameter of >2 cm in the liver, as long as only one of the above four examinations has the typical characteristics of HCC, it can be diagnosed as HCC. 2. For those who have hepatitis B or hepatitis C, or have cirrhosis due to any reason, and identify nodules with a diameter of ≤2 cm in the liver with none or only one of the above four imaging results has typical features of HCC, then liver biopsy can be carried out or close imaging follow-up can be done every 2-3 months. For those who find nodules with a diameter of >2 cm in the liver, and do not have typical features of HCC in the above four imaging tests, then liver biopsy is required for diagnosis. 3. If no intrahepatic nodules are found, AFP levels should be closely followed up as well as imaging examinations every 2-3 months, provided that pregnancy, active liver disease, and gastrointestinal cancer of germinal embryonic origin are ruled out.   **Inclusion Criteria:**   1. Aged between 18 and 70 years old, no gender limit; 2. Patients with primary hepatocellular carcinoma (HCC) who are strictly conformed with the clinical diagnostic criteria of the Guidelines for the Diagnosis and Treatment of Primary Liver Cancer (2022 edition) or diagnosed by histopathology or cytology (patients with fibrolamellar HCC or combined hepatocellular cholangiocarcinoma do not meet the inclusion criteria); 3. Patients have at least one measurable treatment-naive lesion according to the RECIST v1.1 (the long diameter of the measurable lesion on spiral CT scan is ≥10 mm or the short diameter of the malignant lymph node is ≥15 mm); 4. Patients with intermediate-stage primary HCC according to the EASL Clinical Practice Guidelines (version 2020); 5. Barcelona Clinic Liver Stage (BCLC) stage is B; 6. Child-Pugh liver function class A within 7 days prior to initiation of study treatment; 7. Eastern Cooperative Oncology Group (ECOG) performance status score of 0-1 within 7 days prior to initiation of study treatment; 8. Patients were not amenable to curative resection or local ablation; 9. Patients have not received prior anti-tumor treatment with locoregional or systemic therapy; 10. With Expected survival period >3 months; 11. For patient with active hepatitis B virus (HBV) infection, HBV-DNA must be less than 2000 IU/mL (if the research center only has a copy/mL testing unit, it must be less than 12500 copy/mL), and if HBV-DNA ≥2000 IU/mL, the patient should receive anti-HBV treatment according to local standard of care (e.g., entecavir or tenofovir) for a minimum of 14 days before the start of study treatment and are willing to accept antiviral treatment throughout the study period. Hepatitis C virus (HCV) ribonucleic acid (RNA) positive patients must receive anti-HCV therapy per local standard of care (e.g., interferon) and have liver function within common terminology criteria for adverse event (CTCAE) grade 1 elevation; 12. Hematologic and main organ functions are adequate, according to the following laboratory tests within 7 days prior to study treatment: 13. The blood routine examination standards must meet (without blood transfusion within 14 days): hemoglobin (Hb) ≥90 g/L, white blood cell (WBC) count ≥3×10^9^/L, absolute neutrophil count (ANC) ≥1.5×10^9^/L, lymphocyte count ≥0.5×10^9^/L, platelet (PLT) count ≥75×10^9^/L; 14. The biochemical tests should meet the following criteria: alanine aminotransferase (ALT), aspartate aminotransferase (AST), and alkaline phosphatase (ALP) ≤5 times the upper limit of normal (ULN), serum bilirubin (BIL) ≤1.5 times the ULN, serum albumin (ALB) ≥28 g/L, serum creatinine (Cr) ≤1.5 times the ULN or creatinine clearance ≥50 mL/min (calculated by the Cockcroft–Gault equation); 15. The coagulation tests should meet: international normalized ratio (INR) or activated partial thromboplastin time (APTT) ≤2 times the ULN for patients who have not received anticoagulation therapy; 16. The urine routine tests should meet: urine dipstick for proteinuria <2+ (within 7 days prior to initiation of study treatment), patients discovered to have ≥2+ proteinuria on dipstick urinalysis at baseline should undergo a 24-hour urine collection and must demonstrate <1 g of protein in 24 hour; 17. Female patients of childbearing age must have a negative serum pregnancy test (βHCG) or urine HCG test within 7 days before enrollment, and are willing to use appropriate methods of contraception during the trial and 6 months after the last dose of the trial drugs; for males, surgical sterilization, or consent to use an appropriate method of contraception during the trial and 6 months after the last dose of the trial drugs; 18. The patients voluntarily participate in the study, have signed the informed consent form, are able to comply with the study protocol according to the investigator’s judgment, and cooperate with the follow-up.   **Exclusion Criteria:**   1. History of hepatic encephalopathy; 2. Known coagulation disorders and severe blood abnormalities, with severe bleeding tendency. Platelet count <50×10^9^/L and severe coagulation abnormality cannot withstand the study treatment; 3. Untreated or incompletely treated esophageal and/or gastric varices with bleeding or high risk for bleeding (e.g., “red sign” under endoscopy), patients must undergo B-mode US, CT, MRI, or liver elasticity test. All sizes of varices must be assessed and treated per local standard of care (e.g., endoscopic variceal ligation) prior to enrollment. Patients who have underwent the examinations within 6 months prior to the initiation of study treatment do not need to repeat the procedure; 4. A major bleeding due to esophageal and/or gastric varices within 4 weeks before treatment; 5. Refractory massive ascites or cachexia; 6. Severe active infection within 4 weeks prior to initiation of study treatment, including but not limited to hospitalization for complications of infection, bacteremia or severe pneumonia; 7. Have abdominal fistula, gastrointestinal perforation or intra-abdominal abscess within 6 months before study treatment; 8. Treatment with oral or intravenously administered antibiotics within 2 weeks prior to study treatment. Patients receiving prophylactic antibiotics (e.g., for preventing a urinary tract infection) are eligible for this study; 9. Use immunosuppressive or systemic hormone therapy within 2 weeks prior to the initiation of study treatment to achieve the purpose of immunosuppression (e.g., dose >10 mg/day prednisone or other equivalent hormones); 10. Active tuberculosis or positive HIV test at screening; 11. Severe liver, kidney, heart, lung, brain and other major organ dysfunction or failure; 12. Co-infection of HBV and HCV; 13. Patients with high blood pressure that cannot be reduced to normal range after antihypertensive medications (defined as systolic blood pressure >150 mmHg and/or diastolic blood pressure >100 mmHg); 14. Severe cardiovascular disease within 3 months prior to initiation of study treatment, including but not limited to the following diseases: myocardial ischemia or myocardial infarction above grade II, and poorly controlled arrhythmias (including QTc interval ≥ 450 ms for men and ≥ 470 ms for women) or unstable angina, grade III-IV cardiac insufficiency according to the New York Heart Association (NYHA) standard, the left ventricular ejection fraction (LVEF) < 50% indicated by echocardiography, or cerebrovascular accident; 15. History of congenital long QT syndrome or corrected QT interval >500 ms (calculated with the use of the Fridericia method) at screening; 16. Patients with positive urine protein (urinary protein test 2+ or above, or 24-hour urine protein quantitative ≥ 1 g); 17. Serious, non-healing or dehiscing wound, active ulcer, or untreated bone fracture; 18. Uncorrected electrolyte disorder, such as abnormal serum levels of potassium, calcium, or magnesium; 19. Patients who undergo a major surgical approach other than for diagnosis within 4 weeks before study treatment, or anticipation of requirement for a major surgical procedure during the study; 20. History of idiopathic pulmonary fibrosis, organizing pneumonia (e.g., bronchiolitis obliterans), drug-induced pneumonitis, or idiopathic pneumonitis, or evidence of active pneumonitis on screening chest CT scan; 21. Patients with active or history of autoimmune diseases or immune deficiency, including but not limited to the following diseases: myasthenia gravis, myositis, autoimmune hepatitis, systemic lupus erythematosus, rheumatoid arthritis, inflammatory bowel disease, antiphospholipid antibody syndrome, Wegener granulomatosis, Sjögren syndrome, Guillain–Barré syndrome or multiple sclerosis, with the following exception: (1) patients with autoimmune-related hypothyroidism who are on the thyroid replacement hormone; (2) patients with type 1 diabetes mellitus (T1DM) who are on an insulin treatment; (3) patients with eczema, psoriasis, lichen simplex chronicus or vitiligo with dermatological manifestations only; 22. Prior hematopoietic stem cell or solid organ transplantation; 23. History of other malignant tumors within 5 years prior to screening, except for malignancies with a negligible risk of metastasis or death, such as cervical carcinoma in situ, cured skin basal cell carcinoma, localized prostate cancer, ductal carcinoma in situ, or stage I uterine cancer; 24. Treatment with a live, attenuated vaccine within 4 weeks before study treatment, or anticipation of use of such a vaccine during atezolizumab treatment or within 5 months after the last dose of atezolizumab; 25. Previously treated with locoreginal and systemic treatment, such as TACE, selective internal radiation therapy (SIRT) and immunotherapy; 26. Liver function classification is Child-Pugh grade B or C, which cannot be improved by liver protection treatment; 27. Those who have known contraindications, or are hypersensitive/allergic to any drug used in this study previously; 28. Central nervous system (CNS) metastasis; 29. Patients with disturbance of consciousness or unable to comply with the treatment, and patients with mental illness; 30. Patients who have participated in other clinical trials in the past three months; 31. According to the judgment of the investigator, patients with other concomitant diseases that seriously endanger the safety of patients or affect the completion of the study. |
| **Termination Criteria** | **Treatment Termination Criteria:**  Termination of the study treatment does not mean withdrawal from the  study. Participants who terminate study treatment must continue to  complete the remaining study follow-up as required by the protocol.  Participants who meet any of the following criteria, then the study  treatment must be terminated:   1. The participant or his/her legal representative requests to withdraw   from the study;   1. Occurrence of intolerable or unacceptable toxicities associated with the study treatment; 2. Medical imaging or clinical features suggest disease progression; 3. Poor compliance of participants; 4. Another regimen of antitumor therapy that is not associated with the drugs in this study is performed; 5. The participant is pregnant or lactating; 6. Death or loss of follow up; 7. Other situations that the investigators consider necessary for participants to withdraw from the study.   **Withdrawal Criteria:**  Patients must be permanently withdrawn from this study treatment if any of the following occurs:   1. Development of intolerable toxicities related to this study; 2. Presence of a condition that, in the opinion of the investigators, may jeopardize the patient safety if continuation of the study treatment; 3. The investigators believe that withdrawal from the study is in the best interest of the patient; 4. Another treatment regimen that deviates from the treatment protocol has been used; 5. Confirmation of disease progression.   **Research Termination Criteria:**  Criteria for termination in this study include, but are not limited to, the  following:   1. Identification of unintended, meaningful, or unacceptable risks to the patients; 2. Significant failures of the program are identified during the execution of the trial; 3. The study drug/trial treatment is ineffective, or it is pointless to continue the trial; 4. The principal investigator terminates the study. |
| **Drug Delivery**  **Program** | All participants eligible for enrollment into the study will receive  TACE in combination with atezolizumab and bevacizumab as follows:  TACE therapy: conventional TACE was used;  Atezolizumab: 1200 mg, ivgtt, 60 min per infusion (subsequent administration for 30 min if well-tolerated), every 3 weeks as a cycle;  Bevacizumab: 15 mg/kg, ivgtt, 90 min for the initial infusion (subsequent administration for 60 min and 30 min in turn if well-tolerated), every 3 weeks as a cycle.  ✓ TACE was performed within 3 days of enrolment, and if the imaging examination showed dense iodine oil deposition in the tumor foci of the liver, obvious necrosis of the tumor tissues, and there was no enlargement and new foci, close observation could be made, and no re-treatment with TACE is needed. As for the frequency of subsequent TACE treatment, it should be based on the follow-up results, mainly including the patient’s response to the previous treatment (including effectiveness and safety), changes in liver and kidney function and physical status. If untreatable disease progression occurs, TACE therapy should be discontinued. Systemic therapy is repeated every 3 weeks by placing treatment on Day 1 in each cycle with a dosing time window of ±3 days;  ✓ Atezolizumab will be administered by IV infusion at a fixed dose of 1200 mg on Day 1 of each 21-day cycle until unacceptable toxicity as determined by the investigator after an integrated assessment of radiographic and biochemical data, and clinical status of patients. For the first infusion of atezolizumab, atezolizumab (fixed dose of 1200 mg) should be infused for 60 minutes. For the subsequent infusion, atezolizumab should be infused for 30 minutes if the previous infusion was well-tolerated without an infusion-related reaction. If the patient experienced an infusion-related reaction with the previous infusion, the subsequent infusion of atezolizumab should maintain with the initial 60 minutes.  ✓ Bevacizumab will be administered by IV infusion at a dose of 15 mg/kg on Day 1 of each 21-day cycle until unacceptable toxicity as determined by the investigator after an integrated assessment of radiographic and biochemical data, and clinical status of patients. For the first infusion of bevacizumab, bevacizumab (15 mg/kg) should be infused for 90 minutes. For the subsequent infusion, bevacizumab should be infused for 60 minutes and 30 minutes in turn if the previous infusion was well-tolerated without an infusion-related reaction. If the patient experienced an infusion-related reaction with the previous infusion, the subsequent infusion of bevacizumab should maintain with the initial 90 minutes.  ✓ Patients should be informed about the possibility of delayed post-infusion symptoms and instructed to contact their study physician if they develop such symptoms. If the patient experienced an infusion-related reaction with the previous infusion, premedication with antihistamines, antipyretics, and/or analgesics may be administered at the discretion of the investigator.  ✓ No dose modifications for atezolizumab or bevacizumab are allowed. Treatment interruption or permanent discontinuation of atezolizumab or bevacizumab is allowed if clinically indicated.  ✓ Administration of atezolizumab and bevacizumab will be performed in a monitored setting where there is immediate access to trained personnel, adequate equipment and medicine to manage potentially serious adverse events. Vital signs of patients, such as pulse rate, respiratory rate, blood pressure, and temperature, should be measured closely prior to, during and after the infusion.  ✓ Systemic therapy is ongoing until progressive disease, intolerable toxicity, refusal of participant, and other reasons for discontinuation in the opinion of the investigators. |
| **Endpoints** | **Primary Endpoints**  ✓ Objective response rate (ORR) per Response Evaluation Criteria in Solid Tumors (RECIST) v1.1, defined as the percentage of patients with the best response of either complete response (CR) or partial response (PR) ≥ 4 weeks.  **Secondary Endpoints**  ✓ Overall survival (OS), defined as the duration from the initiation of the first study treatment to the date of death from any cause.  ✓ Progression-free survival (PFS), defined as the period from the start of the first study treatment to the date of the first documented tumor progression per RECIST v1.1, appearance of a new lesion, or death due to any cause.  ✓ Objective response rate (ORR) per the Modified Response Evaluation Criteria in Solid Tumors (mRECIST), defined as the percentage of patients with the best response of either complete response (CR) or partial response (PR) ≥ 4 weeks.  ✓ Disease control rate (DCR) per RECIST v1.1 and mRECIST, defined as the percentage of patients who had a CR, PR, or stable disease (SD) for at least 6 months.  ✓ Time to response (TTR) per RECIST v1.1 and mRECIST, defined as the duration from treatment initiation to the first achievement of tumor response (PR or CR).  ✓ Duration of response (DOR) per RECIST v1.1 and mRECIST, defined as duration from the first documented evidence of tumor response (CR or PR) until the first documented disease progression or death from any cause.  ✓ Adverse events (AEs), which was assessed based on National Cancer Institute Common Terminology Criteria for Adverse Events version 5.0 (NCI-CTCAE, v5.0). |
| **Statistical**  **Description** | **Calculation of sample size:**  The sample size calculation was performed by professional statisticians. The sample size was calculated with a one-sided type I error rate (α) of 0.1 and a power (β) of 0.9. The reported ORR of bevacizumab combined with TACE is 35% per RECIST v1.1 in unresectable HCC. We expected that the new therapeutic regimen of TACE combined with atezolizumab plus bevacizumab could increase the ORR from 35% to 55%. Under these assumptions, a sample size of 44 patients should be enrolled. Considering an approximately drop-out rate of 10%, a total sample size of 49 patients was planned for this study.  **Population analysis:**  The full analysis set (FAS) and safety analysis set (SAS), both of which comprised all eligible patients who received at least one session of atezolizumab plus bevacizumab and once TACE, were used to conduct the efficacy and safety assessment, respectively. The patients who did not have at least one post-baseline efficacy assessment will be ruled out from the full analysis set. The safety analysis set will exclude the patients without any safety data.  **Analytical approach:**  Efficacy analysis will be performed based on the whole FAS. The clinical and demographic characteristics of the patients will be summarized with descriptive statistics. Continuous data will be presented as median (interquartile range [IQR] or range). Categorical variables will be summarized as number (percentage). The objective response rate, disease control rate, complete response rate, partial response rate, stable disease rate, progressive disease rate per mRECIST and RECIST v1.1 will be calculated, and the accompanying one-sided 90% confidence intervals (one-sided 90% CIs) will be calculated based on the Clopper-Pearson method. Overall survival and progression-free survival will be estimated using the Kaplan-Meier method, and the corresponding two-sided 95% CIs will be estimated using the Brookmeyer-Crowley method.  Safety analysis will be assessed based on the SAS. The numbers and incidences of each adverse event will be detailed in tabular form.  **Statistical software:**  All statistical analyses were performed using R (version 4.0.2; R Foundation). |
| **Research Period** | Estimated first participant enrollment: August 2021  Expected enrollment of the last participant: December 2021  Expected end of study: December 2022 |

**Research Flowchart**

| **Items** | **Screening period** | | **Treatment period** | | **End of trial** | **Follow-up period** |
| --- | --- | --- | --- | --- | --- | --- |
|  | **Within 4 weeks prior to enrollment** | **Within 1 week prior to enrollment** | **Initial treatment cycle** | **Subsequent treatment cycles** | **Within 4 weeks after checkout** |  |
| **Window period (days)** | **–** | **–** | ± 7 | | | |
| **Baseline information** | | | | | | |
| **Signed informed consent** | **×** |  |  |  |  |  |
| **Demographic information** | **×** |  |  |  |  |  |
| **Past medical history^[1]^** | **×** |  |  |  |  |  |
| **History of tumor treatment^[2]^** | **×** |  |  |  |  |  |
| **Physical exam and vital signs^[3]^** |  | **×** | **×** | Every 3 weeks | **×** |  |
| **Laboratory tests** | | | | | | |
| **Routine blood test^[4]^** |  | **×** | **×** | Every 3 weeks | **×** |  |
| **Urine routine^[5]^** |  | **×** | **×** | Every 3 weeks | **×** |  |
| **Fecal routine^[6]^** |  | **×** | **×** | Every 3 weeks | **×** |  |
| **Blood biochemistry^[7]^** |  | **×** | **×** | Every 3 weeks | **×** |  |
| **Coagulation test^[8]^** |  | **×** | **×** | Every 3 weeks | **×** |  |
| **Thyroid and pituitary function^[9]^** |  | **×** | **×** | Every 3 weeks | **×** |  |
| **Tumor markers^[10]^** |  | **×** | Every 6 weeks | | **×** |  |
| **Infection screening^[11]^** | **×** |  |  |  | **×** |  |
| **Echocardiography^[12]^** | **×** |  |  |  | **×** |  |
| **Electrocardiography^[13]^** |  | **×** | Performed when clinically indicated | | **×** |  |
| **Pregnancy test^[14]^** |  | **×** |  |  | **×** |  |
| **Imaging examination^[15]^** | | | | | | |
| **CT/MRI** | **×** |  | Every 6 weeks | | **×** | **×** |
| **Other clinical assessments and examinations** | | | | | | |
| **ECOG score** |  | **×** | **×** | Every 3 weeks | **×** |  |
| **Adverse events^[16]^** | **×** | **×** | **×** | Every 3 weeks | **×** |  |
| **Other items** | | | | | | |
| **Concomitant medications record^[17]^** | **×** | **×** | **×** | Every 3 weeks | **×** |  |
| **Treatment adherence evaluation^[18]^** | **×** | **×** | **×** | Every 3 weeks |  |  |
| **Follow-up on survival status^[19]^** |  |  |  |  |  | **×** |

**Note:** Perform each examination and procedure strictly according to the timing of the research flowchart, but allow for occasional variations within the window of each examination program (±7 days) due to holidays, vacations, or other administrative reasons.

1. Past medical history: such as history of hypertension, diabetes mellitus, chronic hepatitis B, chronic hepatitis C, cirrhosis, smoke, alcohol drink, drug allergies, lifestyle, the disease staging for initial diagnosis, diagnosis time, etc.
2. History of tumor treatment: including history of surgical treatment, systemic chemotherapy, radiation therapy, ablative therapy, etc. The time of last anti-tumor treatment before signing the informed consent form must be recorded.
3. Physical examination and vital signs: physical examination includes the examination of major body systems (head, face, skin, lymph nodes, eyes, ear, nose and throat, oral cavity, respiratory system, cardiovascular system, abdomen, genitourinary system, musculoskeletal system, nervous system, mental status, etc.). Vital signs include the record of height, weight, body temperature, respiration, blood pressure, and heart rate.
4. Routine blood test: hemoglobin, red blood cell count, white blood cell count, neutrophil count, lymphocyte count, and platelet count within 1 week prior to enrollment, on day 1 of cycle 1 and subsequent cycles (3-week intervals), and at the end of the study.
5. Urine routine: urine protein, urine occult blood, urine PH, urine leukocyte and urine glucose. If semi-quantitative methods show protein ≥2+, 24-hour urine protein quantification is mandatory.
6. Fecal routine: occult blood; If fecal occult blood ≥2+, the results must be reviewed and re-evaluated. When fecal occult blood is confirmed ≥2+, there is a great risk of gastrointestinal bleeding, and gastroscopy is required.
7. Blood biochemistry: including total bilirubin, direct bilirubin, ALT, AST, ALP, LDH, γ-glutamyl transferase, total protein, albumin, urea nitrogen (or urea), creatinine, uric acid, blood glucose, triglycerides, cholesterol, high- and low- density lipoprotein, lipase, amylase, potassium, sodium, chloride, calcium, phosphorus and magnesium.
8. Coagulation test: including prothrombin time (PT), activated partial thromboplastin time (APTT), thromboplastin time (TT), fibrinogen, D-dimer and international normalized ratio (INR).
9. Thyroid and pituitary function: thyroid functional test includes T3, T4, FT3, FT4 and TSH.
10. Tumor markers: AFP and PIVKA-II are tested within 1 week prior to enrollment, on day 1 of two treatment cycles, and at the end of the study.
11. Infection screening: HIV test, detection for five indices of hepatitis B (HBsAg, HBsAb, HBeAg, HBeAb, HBcAb), HCV antibody, HBV-DNA and HCV-RNA quantification are performed within 4 weeks before enrollment and at the end of the study.
12. Echocardiography: performed within 4 weeks prior to enrollment and at the end of the trial. In case of chest pain, palpitation and other symptoms, echocardiography should be additionally performed.
13. Electrocardiography (ECG): performed within 1 week prior to enrollment and at the end of the trial. During the course of the study, additional measurements of ECG are recommended if the patient experiences cardiac discomfort. If symptoms such as precordial pain and palpitations persist, immediate testing of cardiac enzyme profile (e.g., creatine kinase, lactate dehydrogenase), ECG, and additional cardiac ultrasound are recommended.
14. Pregnancy test: limited to women of childbearing age.
15. Imaging examination: including CT or MRI scan of known or suspicious lesions within 4 weeks before enrollment, every 2 treatment cycles during treatment period, and within 4 weeks after the end of trial. The imaging schedule allows for a window of ± 7 days. CT/MRI scans obtained prior to signing informed consent can be used for screening-phase tumor assessment as long as they meet the requirements. Imaging during the treatment period should be performed under the same conditions as the baseline examination (layer thickness of the scan, use of contrast agent, etc.) as far as possible. Imaging examinations will also be required when the participant discontinues the treatment and enters the survival follow-up period, when CT or MRI is performed every 3 months in the first year and every 6 months thereafter.
16. Adverse events: according to NCI-CTCAE v5.0, the observation and recording period of adverse events started from 4 weeks prior to signing the informed consent form until 30 days after the last dose of atezolizumab plus bevacizumab. Until the end of safety follow-up, with recovery of all adverse events to within Grade I or clinical stabilization of all adverse events (whichever is reached later), detailed documentation of adverse events, co-morbid medications/treatments during the treatment period.
17. Concomitant medications record: from 4 weeks prior to signing the informed consent form until the end of the safety follow-up period, collect information on concomitant medications/concomitant therapies (except for solvent medications); record the name of the concomitant medication, the medication dosage, the route of administration, the frequency of administration, the purpose of the administration, and the date of commencement and termination of the medication. Once a participant discontinues trial treatment, concomitant medications or treatments used for new or unresolved adverse events related to trial treatment should be recorded.
18. Treatment adherence evaluation: at the beginning of the first day of each treatment cycle, the medication dose, count and adherence of the previous treatment cycle will be calculated.
19. Follow-up on survival status: after discharge or discontinuation of the study treatment, participants will be regularly followed up by telephone contact or clinic visit, every 3 months in the first year and every 6 months thereafter.

**Abbreviations and Definitions**

| **Abbreviations and special terms** | **Definitions** |
| --- | --- |
| AASLD | The American Association for  the Study of the Liver Disease |
| AE | adverse event |
| AFP | alpha fetoprotein |
| ALB | albumin |
| ALP | alkaline phosphatase |
| ALT | alanine aminotransferase |
| ANC | absolute neutrophil count |
| APTT | activated partial thromboplastin time |
| AST | aspartate aminotransferase |
| BCLC | Barcelona Clinic Liver Cancer |
| BUN | blood urea nitrogen |
| CI | confidence interval |
| CNS | central nervous system |
| Cr | creatinine |
| CR | complete response |
| CRF | case report form |
| CT | computed tomography |
| CTCAE | Common Terminology Criteria for Adverse Event |
| DBil | direct bilirubin |
| DCR | disease control rate |
| DOR | duration of response |
| EASL | the European Association for the Study of the Liver |
| **Abbreviations and special terms** | **Definitions** |
| ECOG PS | Eastern Cooperative Oncology Group  performance status |
| FAS | full analysis set |
| Fbg | fibrinogen |
| GCP | Good Clinical Practice (Code of Practice for Quality Management of  Pharmaceutical Clinical Trials) |
| Hb | hemoglobin |
| HBV | hepatitis B virus |
| HCC | hepatocellular carcinoma |
| HCV | hepatitis C virus |
| HIF | hypoxia-inducible factor |
| HIV | human immunodeficiency virus |
| HR | hazard ratio |
| ICF | informed consent form |
| ICI | Immune checkpoint inhibitor |
| INR | international normalized ratio |
| IQR | interquartile range |
| IRB/IEC | Institutional Review Board/Ethics Committee |
| LDH | lactate dehydrogenase |
| LVEF | left ventricular ejection fraction |
| MDSCs | myeloid-derived suppressor cells |
| mRECIST | the modified Response Evaluation Criteria  in Solid Tumors |
| **Abbreviations and special terms** | **Definitions** |
| MRI | magnetic resonance imaging |
| MWA | microwave ablation |
| NCI-CTCAE | National Cancer Institute Common Terminology Criteria for Adverse Events |
| NA | not available |
| ORR | objective response rate |
| OS | overall survival |
| PD | Progressive disease |
| PD-L1 | programmed cell death 1 ligand 1 |
| PFS | progression-free survival |
| PIVKA-II | protein induced by vitamin K absence-II |
| PLT | platelet count |
| PR | partial response |
| PT | prothrombin time |
| RBC | red blood cell count |
| RECIST | the Response Evaluation Criteria in Solid Tumors |
| RFA | radiofrequency ablation |
| SAE | serious adverse event |
| SD | stable disease |
| SIRT | selective internal radiation therapy |
| SMC | safety monitoring committee |
| SAS | safety analysis set |
| TACE | transcatheter arterial chemoembolization |
| TBil | total bilirubin |
| **Abbreviations and special terms** | **Definitions** |
| TME | Tumor microenvironment |
| TRAE | treatment-related adverse events |
| TT | thromboplastin time |
| TTR | time to response |
| ULN | upper limit of normal value |
| VEGF | vascular endothelial growth factor |
| VEGFR | vascular endothelial growth factor receptor |
| WBC | White blood cell count |

1. **Background and Rationale of the Study**
   1. **Background**
      1. **Epidemiology**

Primary liver cancer is a common malignancy worldwide that includes hepatocellular carcinoma (HCC), intrahepatic cholangiocellular carcinoma (ICC), and combined hepatocellular carcinoma-cholangiocarcinoma (cHCC-CCA), with HCC accounting for approximately 90% of the cases (1). According to GLOBOCAN 2020 data, HCC is the sixth most common cancer and the third leading cause of cancer-related mortality worldwide (2). The etiological factors of HCC mainly include chronic hepatitis B (CHB), chronic hepatitis C (CHC), alcoholic liver disease, and nonalcoholic fatty liver disease (NAFLD) (3).

China has a high incidence rate of HCC, with the incidence and deaths accounting for 45.3% and 47.1% in the world (2). HCC is the fourth most prevalent tumor and the third most lethal tumor in China, with 466,100 patients having newly diagnosed HCC and 422,100 cases mortality due to HCC (4). Among them, HCC ranks third among men and seventh among women. There are more male HCC patients than female HCC patients, with the ratio being 2-3:1. The highest incidence and mortality age of HCC falls on 45-59 years old (4).

- - 1. **Clinical Staging for HCC**

China has a high incidence rate of HCC, accounting for nearly half of the new cases of HCC in the world. Treatment decisions for HCC are made primarily based on tumor burden, liver function and patient’s physical status according to current guidelines (5). Despite well-defined risk factors and established surveillance program in patients with chronic liver disease, 70-80% of patients with HCC are initially diagnosed at an intermediate or advanced stage, which is attributed to the insidious onset of liver cancer, absence of obvious or typical early symptoms and a lack of effective biomarkers for early diagnosis (3, 5). As a result, only 20-30% of HCC patients have access to curative surgery at first presentation, and most of the patients can only receive palliative non-surgical treatment (6).

The Barcelona Clinic Liver Cancer (BCLC) staging system is the most widely used staging system for HCC that is endorsed by the European Association for the Study of the Liver (EASL) and the American Association for the Study of the Liver Disease (AASLD) (5, 7). The BCLC classification divides HCC patients into 5 stages (0, A, B, C and D) based on tumor burden, liver function and patient’s performance status, and is recommended for prognostic prediction and treatment allocation.

- - 1. **Current First-line Treatment for Intermediate HCC**

In BCLC staging system, intermediate HCC (BCLC stage B) is defined as unresectable HCC without vascular invasion or extrahepatic metastasis in a patient with preserved liver function and good performance status. Intermediate HCC constitutes a large subgroup which accounts for 20-30% of all cases (8, 9). The reported median survival of intermediate-stage HCC was 16 months (10, 11), and 49% at 2 years (12). Transarterial chemoembolization (TACE) is the most widely used treatment option for unresectable HCC (13). TACE is also the recommended first line-therapy for patients at intermediate stage of the disease (10, 14). The rationale for TACE for intermediate-stage HCC is that the intra-arterial infusion of a chemotherapeutic agent followed by embolization of the tumor-feeding blood vessels will lead to a strong cytotoxic and ischemic effect, finally resulting in the necrosis and apoptosis of tumor lesions (15, 16). Despite the improvement of patients’ outcomes after the implementation of TACE, the therapeutic effect of TACE monotherapy is not satisfactory. The tumor response rate was only approximately 30% in patients with multinodular or large HCC (17, 18), and the 3-year OS rate was only 26% (19). Furthermore, liver function is a major determinant of survival in HCC patients, and the application scope of TACE is limited by concomitant liver disease and underlying hepatic impairment. Under these circumstances, the patients ultimately progress and are not amenable to further TACE treatment. Therefore, a novel potent and safe treatment modality for intermediate HCC is needed to enhance treatment response and improve the prognosis.

- - 1. **Immunotherapy in the Treatment of HCC**

Immune checkpoint inhibitor (ICI)-based immunotherapy marked a breakthrough and milestone in the treatment of a wide range of cancers, including HCC. ICIs are monoclonal antibodies that enhance the antitumor immune response by blocking the signaling mediated by PD-1 and its ligand like PD-L1 (20). The binding of PD-1 on immune cells to PD-L1 on cancer cells or other cells in the tumor microenvironment (TME) strongly inhibits immune-mediated tumor cell killing. The anti-PD-L1 antibody atezolizumab has shown promising results in phase 1b study of unresectable HCC (21). However, single-agent ICIs only elicited moderate antitumor effect and immune responses.

In this context, ICI-based combination regimens have been investigated to foster synergistic activity and increase the efficacy of ICI in killing HCC. Cotargeting of immune checkpoints and vascular endothelial growth factor (VEGF) recently emerged as a promising treatment strategy. VEGF acts as a strong immunosuppressive molecule by recruitment and activation of immature dendritic cells (DCs), regulatory T cells (Tregs) and myeloid-derived suppressor cells (MDSCs) to the tumor site (22). Moreover, VEGF can inhibit the migration of antitumor lymphocytes via downregulation of adhesion molecules on the neovascular endothelium (23). The anti-VEGF antibody bevacizumab can reverse VEGF-mediated immunosuppression by decreasing the abundance of MDSCs and Tregs and promoting the maturation of DCs (24-27). The phase 3, IMbrave 150 trial showed that in patients with unresectable hepatocellular carcinoma, atezolizumab combined with bevacizumab resulted in better OS and PFS outcomes than sorafenib, with a median PFS of 6.8 months versus 4.3 months (hazard ratio [HR]: 0.59; 95% CI: 0.47-0.76, *P* < 0.001) and a median OS of not reached versus 13.2 months (HR: 0.58; 95% CI: 0.42-0.79, *P* < 0.001) (28). The objective response rates (ORR) were 27.3% (95% CI, 22.5-32.5%) with atezolizumab plus bevacizumab and 11.9% (95% CI, 7.4-18.0%) with sorafenib, according to RECIST v1.1 (*P* < 0.001), and 33.2% (95% CI, 28.1-38.6%) and 13.3% (95% CI, 8.4-19.6%), respectively, according to mRECIST (*P* < 0.001) (28). Based on the positive results, atezolizumab plus bevacizumab is the first immunotherapeutic regimen to receive regulatory approval for the first-line treatment of unresectable HCC.

- - 1. **TACE in Combination with Bevacizumab in HCC**

According to the treatment standards and practice guidelines of HCC, TACE is the first-line recommended treatment for patients with intermediate HCC (10, 14). TACE can directly increase the local concentration of cytotoxic drugs in tumor tissues and enhance the tumor-killing effect of these agents. TACE can also block the blood supply of the tumor, resulting in necrosis and apoptosis of the tumor cells (15, 16, 29). However, TACE-induced ischemia of hypervascularized HCC and surrounding liver parenchyma is accompanied by hypoxia and release of hypoxia-induced factor-1 (HIF-1), leading to a release of proangiogenic cytokines like vascular endothelial growth factor (VEGF) (30-32). High levels of VEGF are associated with a poor prognosis in patients with HCC (33, 34). It is hypothesized that the combined use of the anti-VEGF targeted antibody (e.g., bevacizumab) after TACE may be an effective way to improve the outcomes of HCC patients.

In a pilot study by Britten et al (35), 30 patients with unresectable HCC and Child-Pugh class A or B were randomized in a 1:1 ratio to receive either bevacizumab at a dose of 10 mg/kg of body weight every 14 days beginning 1 week prior to TACE or TACE alone (35). The PFS at 16 weeks was 79% in the TACE-bevacizumab arm versus 19% in the TACE arm (*P* = 0.021). The safety profile was favorable with no life-threatening bevacizumab-related toxicities observed (35). Buijs et al (36). conducted a single-arm phase 2 trial evaluating the safety and efficacy of bevacizumab combined with TACE in patients with HCC at BCLC stage B or C and Child-Pugh score of A or B. Patients were treated with bevacizumab every 2 weeks (10 mg/kg of body weight) and TACE at the third week of a 6-week cycle for up to 3 cycles over 6 months (36). The confirmed ORR and disease control rate (DCR) were 60% and 100%, respectively, according to mRECIST. Bevacizumab-associated severe toxicities included four vascular events (grade 5: duodenal ulcer perforation; grade 3-4: variceal hemorrhage, stroke) and three infections (grade 3-4: cellulitis, cholangitis, clostridium difficile enteritis) (36). Both of the two studies concluded that the combination of TACE and bevacizumab was safe and tolerable in patients with unresectable HCC.

Nevertheless, in a double-blind, phase 2 randomized controlled trial (RCT) by Pinter et al (37), 40 patients with early- or intermediate-stage HCC and Child-Pugh class A or B were scheduled to receive either TACE with bevacizumab (5 mg/kg of body weight every two weeks) or TACE with placebo. After the first TACE procedure, TACE was repeated twice in 4-week intervals if indicated and technically feasible and on demand thereafter. Pinter et al (37). found that radiologic tumor response or OS was not improved in HCC patients who received TACE and bevacizumab, but revealed that severe and even lethal septic and vascular side effects occurred. Thus, the authors advised against bevacizumab as an adjuvant treatment to TACE for HCC patients.

- - 1. **TACE in Combination with Immunotherapy in HCC**

After uptake of tumor antigens by antigen-presenting cells (APCs) and presentation to immature T cells (T-cell priming), activated T cells can recognize the antigens on the tumor cell surface and eliminate cancer cells. Locoregional treatments like TACE and radiofrequency ablation (RFA) can result in immunogenic cell death and release of tumor neoantigens, with subsequent tumor infiltration by APCs and activation of tumor-specific T cells (38-40). However, the limited outcomes of TACE monotherapy show that immune activation via TACE alone may not be sufficient to control tumor growth and prevent HCC recurrence. A combination of TACE and ICI therapy may show synergistic antitumor effects in the treatment of HCC.

Cui et al (41). demonstrated that the combination of RFA and sequential cellular immunotherapy improves PFS for patients with HCC. Wang et al (42). also showed that combination therapy with PD-1 blockade and RFA was superior to RFA alone in improving survival in patients with recurrent HCC. In a pilot study by Duffy et al (43), 39 patients with advanced HCC after progression on sorafenib were treated first with tremelimumab (an anti-CTLA-4 antibody) followed by TACE or RFA targeting one lesion. An encouraging ORR of 26.3% with a median OS and time to progression (TTP) of 12.3 and 7.4 months was observed in this molecular targeted therapy-refractory population (43). No dose-limiting toxicities were encountered. Tumor biopsies in patients with a clinical benefit showed a significant increase and accumulation of intratumoral CD8^+^ T cells (43). All of these clinical studies highlighted the pleiotropic effects of locoregional treatments in modulating tumor microenvironment and strengthens the rationale for developing immunotherapy alongside locoregional treatments.

- 1. **Rationale of the Study**

HCC is a common cancer in China, with high malignant degree and mortality rate. Because of the insidious onset of liver cancer and absence of typical early symptoms, a large proportion of patients with HCC are first diagnosed at relatively late stage, where curative surgery is not feasible. For patients with intermediate-stage HCC, TACE has been established as the standard of treatment. However, the efficacy of TACE monotherapy for intermediate HCC is very limited. The addition of the effective immunotherapeutic regimen of atezolizumab plus bevacizumab to TACE therapy may provide a benefit in patients with intermediate-stage HCC.

Atezolizumab is a monoclonal antibody that targets PD-L1 and blocks the PD-1/PD-L1 interaction (44). TACE can lead to immunogenic cell death and release of tumor neoantigens, which can be recognized by immune-based therapy, like atezolizumab (38). Then, the immune escape mechanisms can be impaired and immune cell functions can be activated (45). Bevacizumab can improve the tumor immune microenvironment by normalizing the tumor vasculature and can counteract against the increased level of proangiogenic cytokine VEGF after TACE.

In the IMbrave150 trial, an ORR of 27.3% by RECIST v.1.1 and 33.2% by HCC-specific mRECIST was reported (28). The median OS was not achieved in the atezolizumab-bevacizumab group, and the OS rates at 6 months and 12 months were 84.8% and 67.2%, respectively. The median PFS was 6.8 months in the atezolizumab-bevacizumab group, and the PFS rate at 6 months was 54.5% (28). However, progressive disease was observed in about 20% of patients with unresectable HCC treated with atezolizumab and bevacizumab (28). Resistance to cancer immunotherapy may be associated with the immunosuppressive factors in the tumor microenvironment. The immunomodulatory effects of TACE on HCC microenvironment could stimulate the antitumor response via tumor antigen release and restore sensitivity to immunotherapy.

To sum up, the preclinical and clinical data presented provide a clear and solid rationale to combine the established standard-of-care TACE with the ICI-based combination regimen atezolizumab plus bevacizumab in patients with intermediate

HCC and to determine its safety and efficacy in a phase 2 clinical trial.

1. **Study Aims and Study Endpoints**
   1. **Primary Research Objectives**

To evaluate the efficacy of TACE combined with atezolizumab plus bevacizumab for intermediate HCC by objective response rate (ORR) per RECIST v1.1.

- 1. **Secondary Research Objectives**

1. Evaluation of the efficacy of TACE combined with atezolizumab plus bevacizumab for intermediate HCC by overall survival (OS);
2. Evaluation of the efficacy of TACE combined with atezolizumab plus bevacizumab for intermediate HCC by progression-free survival (PFS);
3. Evaluation of the efficacy of TACE combined with atezolizumab plus bevacizumab for intermediate HCC by ORR per mRECIST.
4. Evaluation of the efficacy of TACE combined with atezolizumab plus bevacizumab for intermediate-stage HCC by disease control rate (DCR);
5. Evaluation of the efficacy of TACE combined with atezolizumab plus bevacizumab for intermediate-stage HCC by time to response (TTR);
6. Evaluation of the efficacy of TACE combined with atezolizumab plus bevacizumab for intermediate-stage HCC by duration of response (DOR);
7. Evaluation of the safety of TACE combined with atezolizumab plus bevacizumab for intermediate-stage HCC by adverse events (AEs);
   1. **Primary Endpoints**

**✓ Objective response rate (ORR) per RECIST v1.1**

Refers to the proportion of participants with a best overall response of CR or PR as rated according to RECIST v1.1 criteria. If the CR or PR is achieved, participants must be confirmed no less than 4 weeks after the initial evaluation.

Best overall response was defined as the best radiological outcome and was the best outcome documented between the date of first treatment and the date of disease progression according to RECIST v1.1. For patients who have no documented disease progression, the best overall response will be determined based on all efficacy rating.

- 1. **Secondary Endpoints**

**✓ Overall survival (OS)**

Defined as the time interval between the date of the first study treatment and the date of participants’ death due to all causes. Participants who were alive at the last follow-up visit had OS counted as data censored at the time of the last follow-up. The OS of participants who were lost to follow-up was counted as data censored at the time of last confirmed survival.

**✓ Progression-free survival (PFS)**

Defined as the period from the date of the participant’s first study treatment to the date of the first documented tumor progression (as assessed by RECIST v1.1), appearance of a new lesion, or death from any cause, whichever occurred first. Data for patients who were alive and had not experienced disease progression at the last follow-up visit will be censored at the date of the last tumor assessment at which they were known to be progression-free.

**✓ Objective response rate (ORR) per mRECIST**

Refers to the proportion of patients with a best overall response of CR or PR as rated according to mRECIST criteria. If the CR or PR is achieved, participants must be confirmed no less than 4 weeks after the initial evaluation.

**✓ Disease control rate (DCR)**

Refers to the proportion of all participants whose best overall outcome were assessed as complete response (CR), partial response (PR), and stable disease (SD) for at least 6 months according to the RECIST v1.1 or mRECIST criteria.

**✓ Time to response (TTR)**

Defined as the duration from the first study treatment to the first achievement of tumor response on imaging (PR or CR) based on the RECIST v1.1 or mRECIST. For patients who did not achieve PR or CR throughout the treatment period, TTR was not included for analysis.

**✓ Duration of response (DOR)**

Defined as the duration from the first documented evidence of tumor response (CR or PR) until the first documented disease progression according to the RECIST v1.1 or mRECIST criteria, or death due to any cause, whichever occurs first. For patients who did not achieve tumor response during the treatment period, DOR was not included for analysis. Data for patients who were alive and had persistent tumor response throughout the treatment period will be calculated from the first documented evidence of tumor response to the date of the last tumor assessment.

**✓ Adverse events (AEs)**

Adverse events included, but were not limited to, the following: incidence, spectrum, and severity of adverse events (AEs), serious adverse events (SAEs) according to the NCI-CTCAE v5.0 criteria. The number and rate of treatment termination due to adverse events should also be recorded.

1. **Study Design**
   1. **General Design**

This is a single-arm, phase 2 clinical trial to evaluate the efficacy and safety of TACE combined with atezolizumab plus bevacizumab for patients with intermediate-stage hepatocellular carcinoma. Therefore, the data collected and reported in this study will reflect the reality of this triple therapeutic regimen for intermediate HCC.

- 1. **Study Population**
     1. **Sample Size**

According to a previous study, the ORR of bevacizumab combined with TACE as the first-line treatment in unresectable HCC is 35% per RECIST v1.1 criteria (36). We expected that the new therapeutic regimen of TACE combined with atezolizumab plus bevacizumab could increase the ORR from 35% to 55% (H1 = 55%), and a sample size of 44 patients would provide at least 90% power to detect this estimated improvement at a one-sided α level of 10%. Considering an approximately drop-out rate of 10%, a total sample size of 49 patients was planned for this study.

- - 1. **Inclusion Criteria**

1. Aged between 18 and 70 years old, no gender limit;
2. Patients with primary hepatocellular carcinoma (HCC) who are strictly conformed with the clinical diagnostic criteria of the Guidelines for the Diagnosis and Treatment of Primary Liver Cancer (2022 edition) (48), or diagnosed by histopathology or cytology (patients with fibrolamellar HCC or combined hepatocellular cholangiocarcinoma do not meet the inclusion criteria);
3. Patients have at least one measurable treatment-naive lesion according to the RECIST v1.1 (the long diameter of the measurable lesion on spiral CT scan is ≥10 mm or the short diameter of the malignant lymph node is ≥15 mm);
4. Patients with intermediate-stage primary HCC according to the EASL Clinical Practice Guidelines (version 2020);
5. Barcelona Clinic Liver Stage (BCLC) stage is B;
6. Child-Pugh liver function class A within 7 days prior to initiation of study treatment;
7. Eastern Cooperative Oncology Group (ECOG) performance status score of 0-1 within 7 days prior to initiation of study treatment;
8. Patients were not amenable to curative resection or local ablation;
9. Patients have not received prior anti-tumor treatment with locoregional or systemic therapy;
10. With Expected survival period >3 months;
11. For patient with active hepatitis B virus (HBV) infection, HBV-DNA must be less than 2000 IU/mL (if the research center only has a copy/mL testing unit, it must be less than 12500 copy/mL), and if HBV-DNA ≥2000 IU/mL, the patient should receive anti-HBV treatment according to local standard of care (e.g., entecavir or tenofovir) for a minimum of 14 days before the start of study treatment and are willing to accept antiviral treatment throughout the study period. Hepatitis C virus (HCV) ribonucleic acid (RNA) positive patients must receive anti-HCV therapy per local standard of care (e.g., interferon) and have liver function within common terminology criteria for adverse event (CTCAE) grade 1 elevation;
12. Hematologic and main organ functions are adequate, according to the following laboratory tests within 7 days prior to study treatment:
13. The blood routine examination standards must meet (without blood transfusion within 14 days): hemoglobin (Hb) ≥90 g/L, white blood cell (WBC) count ≥3×10^9^/L, absolute neutrophil count (ANC) ≥1.5×10^9^/L, lymphocyte count ≥0.5×10^9^/L, platelet (PLT) count ≥75×10^9^/L;
14. The biochemical tests should meet the following criteria: alanine aminotransferase (ALT), aspartate aminotransferase (AST), and alkaline phosphatase (ALP) ≤5 times the upper limit of normal (ULN), serum bilirubin (BIL) ≤1.5 times the ULN, serum albumin (ALB) ≥28 g/L, serum creatinine (Cr) ≤1.5 times the ULN or creatinine clearance ≥50 mL/min (calculated by the Cockcroft–Gault equation);
15. The coagulation tests should meet: international normalized ratio (INR) or activated partial thromboplastin time (APTT) ≤2 times the ULN for patients who have not received anticoagulation therapy;
16. The urine routine tests should meet: urine dipstick for proteinuria <2+ (within 7 days prior to initiation of study treatment), patients discovered to have ≥2+ proteinuria on dipstick urinalysis at baseline should undergo a 24-hour urine collection and must demonstrate <1 g of protein in 24 hour;
17. Female patients of childbearing age must have a negative serum pregnancy test (βHCG) or urine HCG test within 7 days before enrollment, and are willing to use appropriate methods of contraception during the trial and 6 months after the last dose of the trial drugs; for males, surgical sterilization, or consent to use an appropriate method of contraception during the trial and 6 months after the last dose of the trial drugs;
18. The patients voluntarily participate in the study, have signed the informed consent form, are able to comply with the study protocol according to the investigator’s judgment, and cooperate with the follow-up.
    - 1. **Exclusion Criteria**
19. History of hepatic encephalopathy;
20. Known coagulation disorders and severe blood abnormalities, with severe bleeding tendency. Platelet count <50×10^9^/L and severe coagulation abnormality cannot withstand the study treatment;
21. Untreated or incompletely treated esophageal and/or gastric varices with bleeding or high risk for bleeding (e.g., “red sign” under endoscopy), patients must undergo B-mode US, CT, MRI, or liver elasticity test. All sizes of varices must be assessed and treated per local standard of care (e.g., endoscopic variceal ligation) prior to enrollment. Patients who have underwent the examinations within 6 months prior to the initiation of study treatment do not need to repeat the procedure;
22. A major bleeding due to esophageal and/or gastric varices within 4 weeks before treatment;
23. Refractory massive ascites or cachexia;
24. Severe active infection within 4 weeks prior to initiation of study treatment, including but not limited to hospitalization for complications of infection, bacteremia or severe pneumonia;
25. Have abdominal fistula, gastrointestinal perforation or intra-abdominal abscess within 6 months before study treatment;
26. Treatment with oral or intravenously administered antibiotics within 2 weeks prior to study treatment. Patients receiving prophylactic antibiotics (e.g., for preventing a urinary tract infection) are eligible for this study;
27. Use immunosuppressive or systemic hormone therapy within 2 weeks prior to the initiation of study treatment to achieve the purpose of immunosuppression (e.g., dose >10 mg/day prednisone or other equivalent hormones);
28. Active tuberculosis or positive HIV test at screening;
29. Severe liver, kidney, heart, lung, brain and other major organ dysfunction or failure;
30. Co-infection of HBV and HCV;
31. Patients with high blood pressure that cannot be reduced to normal range after antihypertensive medications (defined as systolic blood pressure >150 mmHg and/or diastolic blood pressure >100 mmHg);
32. Severe cardiovascular disease within 3 months prior to initiation of study treatment, including but not limited to the following diseases: myocardial ischemia or myocardial infarction above grade II, and poorly controlled arrhythmias (including QTc interval ≥ 450 ms for men and ≥ 470 ms for women) or unstable angina, grade III-IV cardiac insufficiency according to the New York Heart Association (NYHA) standard, the left ventricular ejection fraction (LVEF) < 50% indicated by echocardiography, or cerebrovascular accident;
33. History of congenital long QT syndrome or corrected QT interval >500 ms (calculated with the use of the Fridericia method) at screening;
34. Patients with positive urine protein (urinary protein test 2+ or above, or 24-hour urine protein quantitative ≥ 1 g);
35. Serious, non-healing or dehiscing wound, active ulcer, or untreated bone fracture;
36. Uncorrected electrolyte disorder, such as abnormal serum levels of potassium, calcium, or magnesium;
37. Patients who undergo a major surgical approach other than for diagnosis within 4 weeks before study treatment, or anticipation of requirement for a major surgical procedure during the study;
38. History of idiopathic pulmonary fibrosis, organizing pneumonia (e.g., bronchiolitis obliterans), drug-induced pneumonitis, or idiopathic pneumonitis, or evidence of active pneumonitis on screening chest CT scan;
39. Patients with active or history of autoimmune diseases or immune deficiency, including but not limited to the following diseases: myasthenia gravis, myositis, autoimmune hepatitis, systemic lupus erythematosus, rheumatoid arthritis, inflammatory bowel disease, antiphospholipid antibody syndrome, Wegener granulomatosis, Sjögren syndrome, Guillain–Barré syndrome or multiple sclerosis, with the following exception: (1) patients with autoimmune-related hypothyroidism who are on the thyroid replacement hormone; (2) patients with type 1 diabetes mellitus (T1DM) who are on an insulin treatment; (3) patients with eczema, psoriasis, lichen simplex chronicus or vitiligo with dermatological manifestations only;
40. Prior hematopoietic stem cell or solid organ transplantation;
41. History of other malignant tumors within 5 years prior to screening, except for malignancies with a negligible risk of metastasis or death, such as cervical carcinoma in situ, cured skin basal cell carcinoma, localized prostate cancer, ductal carcinoma in situ, or stage I uterine cancer;
42. Treatment with a live, attenuated vaccine within 4 weeks before study treatment, or anticipation of use of such a vaccine during atezolizumab treatment or within 5 months after the last dose of atezolizumab;
43. Previously treated with locoreginal and systemic treatment, such as TACE, selective internal radiation therapy (SIRT) and immunotherapy;
44. Liver function classification is Child-Pugh grade B or C, which cannot be improved by liver protection treatment;
45. Those who have known contraindications, or are hypersensitive/allergic to any drug used in this study previously;
46. Central nervous system (CNS) metastasis;
47. Patients with disturbance of consciousness or unable to comply with the treatment, and patients with mental illness;
48. Patients who have participated in other clinical trials in the past three months;
49. According to the judgment of the investigator, patients with other concomitant diseases that seriously endanger the safety of patients or affect the completion of the study.
    - 1. **Treatment Termination Criteria**

Termination of the study treatment does not mean withdrawal from the study. Participants who terminate study treatment should continue to complete the remaining study follow-up as required by the protocol. Participants who meet any of the following criteria, then the study treatment must be terminated:

1. The participant or his/her legal representative requests to withdraw from the study;
2. Occurrence of intolerable or unacceptable toxicities associated with the study treatment;
3. Medical imaging or clinical features suggest disease progression;
4. Poor compliance of participants;
5. Another regimen of antitumor therapy that is not associated with the drugs in this study is performed;
6. The participant is pregnant or lactating;
7. Death or loss of follow up;
8. Other situations that the investigators consider necessary for participants to withdraw from the study.
   - 1. **Withdrawal Criteria**

Patients must be permanently withdrawn from this study treatment if any of the following occurs:

1. Development of intolerable toxicities related to this study;
2. Presence of a condition that, in the opinion of the investigators, may jeopardize the patient safety if continuation of the study treatment;
3. The investigators believe that withdrawal from the study is in the best interest of the patient;
4. Another treatment regimen that deviates from the treatment protocol has been used;
5. Confirmation of disease progression.
   - 1. **Research Termination Criteria**

Criteria for termination in this study include, but are not limited to, the following:

1. Identification of unintended, meaningful, or unacceptable risks to the patients;
2. Significant failures of the program are identified during the execution of the trial;
3. The study drug/trial treatment is ineffective, or it is pointless to continue the trial;
4. The principal investigator terminates the study.
   - 1. **Lost to Follow-up**

Every possible effort must be made to be informed and report on the participant’s survival status, including contacting the participant. Lost to follow-up is defined as no response to at least 3 contact methods, include, but are not limited to, any of the following: phone calls, faxes, text messages, social media tools, and emails. All attempts to make contact shall be documented on medical record. If it is determined that the participant has died, the research center will use permitted methods to obtain information about the death and the cause of death. The research center may also use public resources, such as community health registries and databases, to obtain contact information. If all attempts have been made and the participant’s survival status cannot be obtained, the investigators should report the date that the participant was last known to be alive and document it in the participant’s medical history.

- - 1. **Definition of end of study**

Twelve months after the first cycle of treatment in the final case, the study is completed and will be statistically analyzed for primary and secondary endpoints of the study, and additional analyses of the study’s primary and secondary endpoints will be performed at the end of follow-up. The occurrence of serious adverse events (SAEs) will be collected and documented during the treatment period and after the final dose, as specified in the protocol.

- 1. **Drug Delivery Programs**

All participants eligible for enrollment into the study will receive TACE in combination with atezolizumab and bevacizumab as follows:

✓ TACE therapy: conventional TACE was used;

✓ Atezolizumab: 1200 mg, ivgtt, 60 min per infusion (subsequent administration for 30 min if well-tolerated), every 3 weeks as a cycle;

✓ Bevacizumab: 15 mg/kg, ivgtt, 90 min for the initial infusion (subsequent administration for 60 min and 30 min in turn if well-tolerated), every 3 weeks as a cycle.

✓ TACE was performed within 3 days of patient enrolment, and if the imaging examination showed dense iodine oil deposition in the tumor foci of the liver, obvious necrosis of the tumor tissues, and there was no enlargement and new foci, close observation could be made, and no re-treatment with TACE is needed. As for the frequency of subsequent TACE treatment, it should be based on the follow-up results, mainly including the patient’s response to the previous treatment (including effectiveness and safety), changes in liver and kidney function and physical status. If untreatable disease progression occurs, TACE therapy should be discontinued. Systemic therapy is repeated every 3 weeks by placing treatment on Day 1 in each cycle with a dosing time window of ±3 days;

✓ Atezolizumab will be administered by IV infusion at a fixed dose of 1200 mg on Day 1 of each 21-day cycle until unacceptable toxicity as determined by the investigator after an integrated assessment of radiographic and biochemical data, and clinical status of patients. For the first infusion of atezolizumab, atezolizumab (fixed dose of 1200 mg) should be infused for 60 minutes. For the subsequent infusion, atezolizumab should be infused for 30 minutes if the previous infusion was well-tolerated without an infusion-related reaction. If the patient experienced an infusion-related reaction with the previous infusion, the subsequent infusion of atezolizumab should maintain with the initial 60 minutes.

✓ Bevacizumab will be administered by IV infusion at a dose of 15 mg/kg on Day 1 of each 21-day cycle until unacceptable toxicity as determined by the investigator after an integrated assessment of radiographic and biochemical data, and clinical status of patients. For the first infusion of bevacizumab, bevacizumab (15 mg/kg) should be infused for 90 minutes. For the subsequent infusion, bevacizumab should be infused for 60 minutes and 30 minutes in turn if the previous infusion was well-tolerated without an infusion-related reaction. If the patient experienced an infusion-related reaction with the previous infusion, the subsequent infusion of bevacizumab should maintain with the initial 90 minutes.

✓ Patients should be informed about the possibility of delayed post-infusion symptoms and instructed to contact their study physician if they develop such symptoms. If the patient experienced an infusion-related reaction with the previous infusion, premedication with antihistamines, antipyretics, and/or analgesics may be administered at the discretion of the investigator.

✓ No dose modifications for atezolizumab or bevacizumab are allowed. Treatment interruption or permanent discontinuation of atezolizumab or bevacizumab is allowed if clinically indicated.

✓ Administration of atezolizumab and bevacizumab will be performed in a monitored setting where there is immediate access to trained personnel, adequate equipment and medicine to manage potentially serious adverse events. Vital signs of patients, such as pulse rate, respiratory rate, blood pressure, and temperature, should be measured closely prior to, during and after the infusion.

✓ Systemic therapy is ongoing until progressive disease, intolerable toxicity, refusal of participant, and other reasons for discontinuation in the opinion of the investigators.

- 1. **Duration of the Study**

The study is expected to last 16 months, including a 4-month enrollment period.

⚫ Date of enrollment/start of study for first patient: August 2021

⚫ Date of last patient enrollment: December 2021

⚫ Study end date: December 2022

⚫ Database lock date: August 2022

⚫ Reporting date: October 2022

Note: If the actual duration of the study changes, it is not a violation of the program.

- 1. **Dispensing, Storage, Administration and Recall of Medicines**

The distribution of drugs should be strictly managed. The center should have a person in charge of issuing and keeping the drugs, and the researcher should fill in the detailed records of drug collection and use at each visit.

According to the requirements of GCP, the study drugs are kept, issued and recalled by the test unit. Test drugs should be kept according to the instructions, and the expiration date is detailed in the instructions.

The investigator or study authorized personnel shall record the date and amount of medication administered to each participant separately. Remaining test medications are inventoried and periodically recycled for destruction. If destruction is required at the research center, the investigator must ensure that the destruction complies with applicable environmental regulations, unit policies, and other applicable provisions and provide the relevant processes for destruction. Records should be kept of all destruction.

1. **Research Process**
   1. **Screening Period**

Unless otherwise noted, it is recommended that the following participant screening steps be completed within 4 weeks prior to initiation of study drug therapy:

⚫ Demographic information: names, gender, ethnicity, marital status, date of birth, height, weight, etc.

⚫ Past medical history: history of hypertension, diabetes mellitus, chronic hepatitis B, chronic hepatitis C, cirrhosis, smoke, alcohol drink, drug allergies, lifestyle, etc.

⚫ Tumor diagnosis: clinical and pathological diagnosis, tumor stage, etc.

⚫ History of tumor treatment: history of surgical treatment, systemic chemotherapy, radiation therapy, ablative therapy, etc.

 ✧ Tumor surgical treatment: date of surgery, name of surgery; surgical procedure, surgical grade, etc.

✧ Tumor chemotherapy: chemotherapeutic agents, medication administration, dosage, frequency, efficacy, date of chemotherapy, etc.

✧ Tumor radiotherapy: date of radiotherapy, radiation dose and mode, etc.

✧ Tumor ablative therapy: ablation date, ablation method and efficacy, etc.

⚫ Infection screening: HIV test, detection for five indices of hepatitis B (HBsAg, HBsAb, HBeAg, HBeAb, HBcAb), HCV antibody, HBV-DNA and HCV-RNA quantification, etc.

⚫ Echocardiography: In case of chest pain, palpitation and other symptoms, echocardiography should be additionally performed.

⚫ Tumor imaging: including CT or MRI scan of known or suspicious lesions. Imaging during the treatment period should be performed under the same conditions as the baseline examination (layer thickness of the scan, use of contrast agent, etc.) as far as possible.

After the patient is admitted to the hospital, it is determined whether the patient meets the enrollment criteria. For patients who meet the enrollment criteria, the physician introduces the details of the trial and the patient agrees to sign the informed Consent Form.

The following screening steps were completed within 1 week prior to initiation of study treatment:

⚫ Physical examination: height, weight, head, face, skin, lymph nodes, eyes, ear, nose and throat, oral cavity, respiratory system, cardiovascular system, abdomen, genitourinary system, musculoskeletal system, nervous system, mental status, etc.

⚫ Vital signs: heart rate, respiratory rate, temperature, blood pressure.

⚫ Routine blood test: hemoglobin, red blood cell, white blood cell, neutrophil count, lymphocyte count, and platelet count.

⚫ Urine routine: urine protein, urine occult blood, urine PH, urine leukocyte and urine glucose. If semi-quantitative methods show protein ≥2+ (e.g., urine test strips), 24-hour urine protein quantification is performed. Enrolled patients must have a 24-hour urine protein quantification of <1g.

⚫ Fecal routine: fecal occult blood. If fecal occult blood ≥2+, the results must be reviewed and re-evaluated. When fecal occult blood is confirmed ≥2+, there is a great risk of gastrointestinal bleeding, and gastroscopy is required.

⚫ Blood biochemistry: total bilirubin, direct bilirubin, ALT, AST, ALP, LDH, γ-glutamyl transferase, total protein, albumin, urea nitrogen (or urea), creatinine, uric acid, blood glucose, triglycerides, cholesterol, high- and low- density lipoprotein, lipase, amylase, potassium, sodium, chloride, calcium, phosphorus and magnesium.

⚫ Coagulation test: prothrombin time, activated partial thromboplastin time, thromboplastin time, fibrinogen, D-dimer and international normalized ratio.

⚫ Thyroid and pituitary function: thyroid functional test includes thyrotropin, triiodothyronine, free triiodothyronine, thyroxine, free thyroxine, etc.

⚫ Tumor markers: alpha-fetoprotein (AFP) and protein induced by vitamin K absence or antagonist-II (PIVKAII).

⚫ ECG: 12-lead electrocardiogram including heart rate, QT interval and QTc

interval.

⚫ Pregnancy test: human chorionic gonadotophin (HCG) test, which is limited to women of childbearing age.

⚫ ECOG performance status score.

Upon completion of all of the above screening assessments, eligible patients will enter the study treatment period.

- 1. **Treatment Period**

The treatment period is calculated from the start of enrolled patients receiving treatment, and TACE must be administered within 3 days of enrollment. If contrast-enhanced computed tomography (CT) or magnetic resonance imaging (MRI) reveals dense iodine oil deposition, obvious necrosis of the tumor, and no enlargement or new lesions, re-treatment with TACE is not considered. The frequency of TACE was determined by the follow-up results during the treatment period.

Systemic treatment will be initiated 2-14 days after the first session of TACE when liver function of patients recovered to normal. The treatment cycle is calculated from the time the participant receives atezolizumab plus bevacizumab and is administered in cycles every 21 days. On the first day of each cycle, atezolizumab will be administered intravenously at a fixed dose of 1200 mg (initial intravenous injection for 60 min, and subsequent intravenous injections for 30 min if well-tolerated). Bevacizumab will be intravenously infused at a dose of 15 mg/kg at least 5 min apart (initial intravenous injection was for 90 min, then 60 min and then 30 min in turn if well-tolerated). Systemic treatment would continue until progressive disease, intolerable toxicity, refusal of treatment, and other conditions that the investigators considered necessary to discontinue treatment. Dose modifications of atezolizumab and bevacizumab will not be allowed, while permanent discontinuation of one study drug was allowed if clinically indicated.

During treatment period, patients completed different examinations on day 1 of each systemic treatment cycle every 3 weeks, including physical examination, vital signs, Eastern Cooperative Oncology Group performance status (ECOG-PS) score, and laboratory tests. The safety and adverse events are also monitored every 3 weeks during treatment period. Participants also need to complete tumor imaging evaluation (evaluation method is consistent before and after, CT or MRI) at a frequency of one time every 6 weeks (±7 days) after the first administration of the drugs.

- 1. **Follow-up Period**

Participants are determined to enter the follow-up period on the day after the last dose prior to discharge from the group and until completion of the survival follow-up:

⚫ Safety follow-up: Adverse events that do not recover after discontinuation of the

treatment should be followed up. Participants are followed for safety for 90 days

after their last cycle of systemic treatment for adverse events, concomitant medications, treatment compliance, dispensing and recovery of drugs.

⚫ Efficacy follow-up: For participants who are discharged from the study for reasons other than disease progression, unacceptable toxicities, or death, tumor imaging evaluations will continue to be performed as originally planned, with the frequency of evaluations as previously planned (one evaluation every 6 weeks) until progressive disease, initiation of other antitumor agents, lost to follow-up, or death. The duration of each follow-up visit, results of tumor imaging evaluations, and other antitumor therapy information should be recorded in detail.

⚫ Survival follow-up: All non-dead participants who completed safety follow-up and efficacy follow-up (whichever was completed later) underwent survival follow-up. Survival data (date of death and cause of death) and post-research treatment will be collected by telephone interviews every 3 months during the first year of the follow-up period and every 6 months thereafter, until death, lost to follow-up or the study is terminated. Each survival follow-up visit is to be documented in detail on a follow-up form.

1. **Adverse Events Reporting**
   1. **Adverse Events**
      1. **Definition of adverse events**

Adverse events (AEs) are any adverse medical events that occur after a clinical trial participant receives treatments, but is not necessarily causally related to the treatment. AEs can be any unfavorable and undesired sign, symptom, abnormal laboratory test, or disease, etc., and includes at least the following:

1) An exacerbation of a pre-existing medical condition/disease (including an

exacerbation of signs, symptoms, or laboratory abnormalities) prior to entry into

the clinical trial;

2) Any new adverse medical condition (including signs, symptoms, and new

illnesses) during the clinical trial;

1. Clinically significant abnormal laboratory results.

The investigator should keep a detailed record of any AEs that occurs in the participant, including: the name of the AEs and a description of all associated symptoms, time of occurrence, severity, relevance to the trial medication, duration, measures taken to treat AEs, final results and regression.

- - 1. **Criteria for determining the severity of AE**

Refer to NCI-CTCAE v5.0 for grading criteria for drug AEs. Grade refers to the severity of the AE. The CTCAE displays Grades 1 through 5 with unique clinical descriptions of severity for each AE:

| **Grade** | **Clinical description of severity** |
| --- | --- |
| 1 | Mild: asymptomatic or mild symptoms; clinical or diagnostic observations only; intervention not indicated. |
| 2 | Moderate: minimal, local or noninvasive intervention indicated; limiting age-appropriate instrumental Activities of Daily Living (ADL). Age-appropriate ADL with tools means cooking, shopping, talking on the phone, counting money, etc. |
| 3 | A condition that is severe or medically significant but is not immediately life-threatening; hospitalization or prolongation of hospitalization indicated; disabling; and limiting self-care ADL. Self-care ADLs are: bathing, dressing, undressing, eating, going to the bathroom, taking medication, etc., and are not bedridden. |
| 4 | Life-threatening consequences; urgent medical intervention required. |
| 5 | Death related to AE. |

- - 1. **Determination of the relationship between AEs and the treatments**

The collection of AEs starts from the signing of the informed consent, regardless of whether the events are related to the treatments or not, and even regardless of whether the treatments are started or not. During the treatment period, any complaints of discomfort or abnormal changes in objective laboratory tests should be recorded, and the severity, duration, treatment and regression of the AEs should be noted. The investigator should comprehensively determine the relationship between the AEs and the treatments. Possible associations between the AEs and the treatments are evaluated as "related" or "unrelated".

Relevant: the AEs is reasonably related to the treatments and is medically (pharmacologically or clinically) attributable to the treatments.

Unrelated: AEs lacks reasonable correlation with the treatments, cannot be medically (pharmacologically or clinically) attributed to the treatments, and/or is explained by other plausible reasons, e.g., underlying disease, comorbidities, coadministration of medications.

- 1. **Serious Adverse Events**
     1. **Definition of serious adverse events**

Serious adverse events (SAEs) are medical events that occur during a clinical trial that require hospitalization or prolonged hospitalization, are disabling, have limited self-care activities of daily living (ADL), are life-threatening or fatal, or result in congenital malformations. It includes the following medical events:

⚫ Events resulting in mortality;

⚫ Life-threatening consequences (defined as the participant being in immediate danger of death and urgent medical intervention needed at the time of the events);

⚫ Events requiring hospitalization or prolongation of hospitalization;

⚫ Events that can result in permanent or severe disability/malfunction/impairment of

ability to self-care ADL (Self-care ADL included, but not limited to, bathing, dressing, undressing, eating, going to the bathroom, taking medication);

⚫ Congenital anomalies or birth defects;

⚫ Other medically significant events.

- - 1. **Hospitalization**

AEs that result in hospitalization (even if <24h) or prolonged hospitalization in clinical trials should be considered as SAEs. Hospitalization does not include the following:

⚫ Nursing homes;

⚫ Rehabilitation organization;

⚫ Routine emergency room admission (<24h);

⚫ Day surgery (e.g., outpatient/same-day/ambulatory);

⚫ Social reasons (health insurance reimbursement, etc.).

Hospitalization or prolonged hospitalization which is not associated with the worsening of an AE should not be considered as SAE. e.g.:

⚫ Hospitalization for management reasons (e.g., routine annual physicals);

⚫ Hospitalization as defined by the trial protocol during the clinical trial (e.g., as

required by the trial protocol);

⚫ Elective hospitalization not related to worsening AE (e.g., elective surgery);

⚫ Admission to the hospital for a pre-existing condition without the development of a new AE or an exacerbation of the pre-existing condition (e.g., in order to check for laboratory test abnormalities that have persisted to date prior to the test);

⚫ Scheduled treatments or surgical procedures should be documented throughout the trial protocol and/or in the individual participant's baseline information;

⚫ Admission for blood product use only.

Diagnostic or therapeutic invasive (e.g., surgery), noninvasive procedures should not be reported as an AE, but the disease condition that led to the procedure should be reported if it meets the definition of an AE, e.g., acute appendicitis that develops during the AE reporting period should be reported as an AE, and the appendectomy that is performed as a result of it should be documented as the treatment for that AE.

- - 1. **Disease progression and death**

Disease progression is defined as deterioration of the participant’s condition caused by HCC, including imaging progression and progression of clinical signs and symptoms. New metastases from the primary tumor is considered as disease progression. Events that are life-threatening, require hospitalization or prolonged hospitalization, or result in permanent or severe disability/insufficiency/impairment of ability to self-care ADL, congenital anomalies, or birth defects due to disease progression are not reported as SAEs. If there is any uncertainty as to whether the SAE is due to disease progression, it should be reported as an SAE.

In this study population, disease progression is an expected occurrence and the term “disease progression” cannot be reported as an AE. When disease progression occurs, the event used to identify disease progression should be reported as an AE. e.g., a participant develops epilepsy that is determined to be related to brain metastases, and the AE terminology should be documented as “epilepsy” rather than “disease progression” or “brain metastases”.

If a participant dies during the trial, it must be reported as an SAE, regardless of whether or not new antitumor therapy has been received. Deaths evaluated by the investigator as likely to be attributed to signs and symptoms of disease progression should be recorded in the eCRF and reported as an SAE. The term “death” should not be used as an AE or SAE term, but rather as the result of an event, and the event that caused or contributed to the death should be recorded as an AE or SAE. If the cause of death is unknown and cannot be determined at the time of reporting, the AE or SAE terminology should be recorded as “unexplained death”.

- 1. **Pathways of Adverse Events Recording and Reporting**

Clinical adverse events may occur during the treatment of the participant. Once the adverse events (including serious adverse events) occur, they should be recorded in detail the time of occurrence, clinical manifestations, treatment and duration, regression, and the relationship with the drugs and treatments. Patients presenting with abnormal laboratory tests need to be followed until the test results return to normal, or to pre-treatment levels, or are determined to be unrelated to the treatment.

SAEs should be collected from participant enrollment until the 90 days after the last treatment cycle. In case of SAE, whether it is the first report or follow-up report, the investigator of each participating center must immediately fill in the Serious Adverse Event Reporting Form, sign and date it, and immediately notify the the independent Data and Safety Monitoring Committee within 24 hours of the investigator’s notification, and report it to the relevant units in accordance with the regulatory requirements, and the specific contact person information for SAE reporting is detailed in the table below.

| **Unit (of measure)** | **Department contacted** | **Reporting modalities** | **Contact details** |
| --- | --- | --- | --- |
| State Food and Drug Administration (SFDA) | Drug Research Supervision Division, Department of  Drug and  Cosmetic  Registration and  Administration | Fax is preferred.  In the event that faxing is not  possible, mail by EMS (if  mailing by EMS, please keep a  record of the fax failure and  keep a copy of the EMS  receipt). | Tel: 010-68313344-1003  Fax: 010-88363228  Address: Building 2, No.26  Xuanwumen West Street, Xicheng District, Beijing, China (100053) |
| National Health and  Family Planning  Commission | Medical and  Health Bureau  (MHB) | Fax is preferred.  In the event that faxing is not  possible, mail by EMS (if  mailing by EMS, please keep a  record of the fax failure and  keep a copy of the EMS  receipt). | Tel: 010-68792201  Fax: 010-68792734  Address: No.A38, Beilishi  Road, Xicheng District,  Beijing, 100810, P.R. China |
| China Registered  Clinical Trial  Center | Ethics Committee | Email | Mail: chic ictr@vip.qq.com  Address: Room 2092, 2/F,  Octagonal Pavilion,  Administration Building,  West China Hospital,  Sichuan University, 37  Guoxue Lane, Chengdu,  Sichuan, 610041, P.R. China |
| Provinces,  autonomous  regions, municipalities directly under the  Central Drug  Administration | Refer to the provinces, autonomous regions, municipalities directly under the Central Drug Administration requirements. | | |

SAEs should be documented in detail with regard to symptoms, severity, relevance to the trial drug, time of onset, time of management, measures taken, time and mode of follow-up, and regression. If an SAE is not considered by the investigator to be related to the test drug, but is potentially related to a study condition (e.g., termination of the original treatment, or comorbidities during the course of the trial), this relationship should be detailed in the narrative portion of the SAE report form. If the intensity of an ongoing SAE or its relationship to the test drug changes, a follow-up report should be submitted immediately. If the investigator believes that information was misreported in a previously reported SAE, a correction, withdrawal, or downgrading statement may be made in the follow-up report and reported in accordance with the SAE reporting procedures.

- 1. **Risk Prevention and Treatment**

Participants should be closely monitored during the course of treatment, and adverse events or complications should be immediately treated when detected.

1. **Data Collection Plan and Statistical Analysis**
   1. **Data Collection Plan**

The duration of data collection is from patient enrollment until 12 months after the end of the last dose of treatment, and all adverse events were recorded in the CRF.

Each patient will receive a scheduled visit and specific data will be recorded at various time points during the visit.

All examinations are recommended and specific examinations are based on actual clinical practice.

- 1. **Statistical Analysis of Data Sets**

Full analysis set (FAS): the efficacy of all cases in which the treatments are used at least once is analyzed according to the principle of intention-to-treat (ITT). For cases where the full course of treatment is not observed, the last observation data is used as the final outcome of the trial.

Safety analysis set (SAS): all patients who has received treatment at least once and has a post-dose safety record are included in the safety analysis set.

- 1. **Statistical Analysis Plan**

Continuous data were summarized as median (interquartile range [IQR] or range). Categorical data were summarized as number (percentage). The percentages and one-sided 90% confidence intervals (CIs) for ORR and DCR were estimated using the Clopper-Pearson method. Survival was estimated using the Kaplan-Meier method, and the corresponding two-sided 95% CIs were estimated using the Brookmeyer-Crowley method. All statistical analyses were performed using R (version 4.0.2; R Foundation).

- 1. **Basic Patient Characteristics**

Median, maximum, minimum, upper quartile, lower quartile values are calculated for quantitative data such as age and tumor size, and number and percentage are listed for qualitative data such as ECOG-PS score and Child-Pugh grade.

- 1. **Efficacy Analysis**

Primary efficacy indicators: Evaluating objective response rate (ORR, CR+PR) according to the RECIST v1.1 criteria and the one-sided 90% CI.

Secondary efficacy indicators: Evaluating ORR according to the mRECIST criteria and the one-sided 90% CI. Assessing disease control rate (DCR, CR+PR+SD) based on the RECIST v1.1 and mRECIST criteria, respectively, and their corresponding one-sided 90% CI. Evaluating median time to response (TTR) and median duration of response (DOR) and their upper and lower quartile values, respectively, according to the RECIST v1.1 and mRECIST criteria. Plotting Kaplan-Meier survival curves of OS and PFS and estimating median OS and median PFS and their two-sided 95% CI.

- 1. **Safety Analysis**

Adverse events occurred in this study are described in a table based on descriptive statistical analysis (The relationship of the adverse events to the study drug was recorded as “definitely related/ probably related/ possibly related”). Adverse events were described as grades and percentages of patients.

1. **Data Management**

Appropriate training will be provided to the investigator and authorized research staff prior to the start-up of the study center. Data entry should be completed during or as soon as possible after the visit and kept up-to-date to ensure that it reflects the latest developments in the participants enrolled in the study. To avoid discrepancies in the assessment of outcomes by different assessors, it is recommended that baseline and all subsequent efficacy and safety assessments be completed by the same person for the same participant. The investigator is required to review the data to ensure the accuracy and correctness of all data entered into the data collection form. If certain assessments were not performed during the course of the study, or if certain information is not available, applicable, or unknown, the investigator should record it on the data collection form.

Documents (protocols, etc.) from clinical trials need to be kept and managed in accordance with GCP requirements. Research centers should keep these documents until 5 years after the end of the study. Study documents should be reasonably preserved for future access or data traceability. Security and environmental risk issues should be considered when preserving documents.

1. **Quality Control and Quality Assurance**

Investigators must be physicians trained in clinical trials and work under the supervision of a senior professional. Pre-test inspection of clinical wards must be in accordance with standardized requirements to ensure that resuscitation equipment is available. It is recommended that a professional caregiver administer the medication to the participant, with detailed information on how the medication is being taken, to ensure compliance. The research center must strictly follow the study protocol and faithfully fill in the case report form.

Supervisors should follow standard operating procedures to oversee the conduct of the clinical trial, confirm that all data are recorded and reported correctly and completely, that all case report forms are entered correctly and are consistent with the original information, and ensure that the trial is performed in accordance with the clinical study protocol.

1. **Ethical, Regulatory and Administrative Principles**
   1. **Local Legislation and Declaration of Helsinki**

The investigator ensures that the study is conducted in full compliance with the principles of the Declaration of Helsinki or the laws of the country in which the study is being conducted, regardless of the country’s provisions for the protection of human rights. The study must be conducted in strict compliance with the Guideline for Good Clinical Practice ICH Harmonized Tripartite Guideline, or the local laws, whichever is more stringent.

- 1. **Informed Consent**

It is the responsibility of the investigator or his/her delegate (if permitted by local law) to explain in detail to each participant who participates in the trial the purpose of the trial, the methodology, the benefits of participation and the potential hazards, and then to obtain a written informed consent form signed by each participant participating in the study. For participants who are unable to sign the informed consent, the consent form must be signed by their legal representative. If neither the participant nor his/her legal representative is capable of reading, a notary public should be present throughout the informational process. After the participant and his/her legal representative have given verbal consent to participate in the trial, the notary signs the informed consent form, certifying that its contents have been accurately interpreted and understood. The investigator or principal investigator must also state that the participant has the full right to refuse to participate in the trial or to discontinue the trial at any time for any reason.

- 1. **Independent Ethics Committee and Statutory Audit Committee**

The protocol and related materials provided to the participants should be submitted by the investigator to an independent ethics committee. The approval of the committee must be obtained to initiate the trial, indicating the date on which the committee met to discuss and approve it. After obtaining approval from the independent ethics committee, any changes to the protocol must be submitted by the investigator to the committee in accordance with local procedures and regulatory requirements. If there is no local accreditation authority, the researcher should submit the protocol to the regional committee. If there is no regional committee, the investigator should submit the protocol to the European Ethical Review Committee.

- 1. **Confidentiality Agreements**

The investigator or any member of his/her team shall not disclose such material or information to unauthorized persons without the prior formal written consent of the principal investigator.

Except for information permitted to be disclosed by statute, the investigator shall maintain the confidentiality of all information received, obtained, or derived in the course of this research, and shall take all steps necessary to ensure that there is no breach of confidentiality.

- 1. **Record Keeping**

The investigator should arrange for the custody of study documents until the end of the study. In addition, with regard to patient record keeping, the investigator should comply with specific local regulations/guidelines.

Unless otherwise stated in the investigator’s protocol, it is recommended that the investigator keep the study documentation until at least five years after completion or discontinuation of the study, in accordance with other standards and/or local laws.

- 1. **Early Discontinuation of Research**

The principal investigator of the sponsoring organization may decide to discontinue the study at any time and for any reason. The decision to discontinue the study will be communicated in writing to the participating researchers. Similarly, if another investigator decides to withdraw from the study, he/she must inform the principal investigator in writing.

**References**

1. Llovet JM, Kelley RK, Villanueva A, Singal AG, Pikarsky E, Roayaie S, et al. Hepatocellular carcinoma. Nat Rev Dis Primers. 2021;7(1):6.

2. Sung H, Ferlay J, Siegel RL, Laversanne M, Soerjomataram I, Jemal A, et al. Global Cancer Statistics 2020: GLOBOCAN Estimates of Incidence and Mortality Worldwide for 36 Cancers in 185 Countries. CA Cancer J Clin. 2021;71(3):209-49.

3. Villanueva A. Hepatocellular Carcinoma. N Engl J Med. 2019;380(15):1450-62.

4. Chen W, Zheng R, Baade PD, Zhang S, Zeng H, Bray F, et al. Cancer statistics in China, 2015. CA Cancer J Clin. 2016;66(2):115-32.

5. EASL Clinical Practice Guidelines: Management of hepatocellular carcinoma. J Hepatol. 2018;69(1):182-236.

6. Forner A, Reig M, Bruix J. Hepatocellular carcinoma. Lancet. 2018;391(10127):1301-14.

7. Heimbach JK, Kulik LM, Finn RS, Sirlin CB, Abecassis MM, Roberts LR, et al. AASLD guidelines for the treatment of hepatocellular carcinoma. Hepatology. 2018;67(1):358-80.

8. Cillo U, Vitale A, Grigoletto F, Farinati F, Brolese A, Zanus G, et al. Prospective validation of the Barcelona Clinic Liver Cancer staging system. J Hepatol. 2006;44(4):723-31.

9. Goh GB, Li JW, Chang PE, Chow KY, Tan CK. Deciphering the epidemiology of hepatocellular carcinoma through the passage of time: A study of 1,401 patients across 3 decades. Hepatol Commun. 2017;1(6):564-71.

10. Llovet JM, Bruix J. Systematic review of randomized trials for unresectable hepatocellular carcinoma: Chemoembolization improves survival. Hepatology. 2003;37(2):429-42.

11. Llovet JM, Bruix J. Molecular targeted therapies in hepatocellular carcinoma. Hepatology. 2008;48(4):1312-27.

12. Cabibbo G, Enea M, Attanasio M, Bruix J, Craxì A, Cammà C. A meta-analysis of survival rates of untreated patients in randomized clinical trials of hepatocellular carcinoma. Hepatology. 2010;51(4):1274-83.

13. Takayasu K, Arii S, Ikai I, Omata M, Okita K, Ichida T, et al. Prospective cohort study of transarterial chemoembolization for unresectable hepatocellular carcinoma in 8510 patients. Gastroenterology. 2006;131(2):461-9.

14. Bruix J, Sherman M. Management of hepatocellular carcinoma: an update. Hepatology. 2011;53(3):1020-2.

15. Chen S, Yu W, Zhang K, Liu W. Comparison of the efficacy and safety of Transarterial chemoembolization with and without Apatinib for the treatment of BCLC stage C hepatocellular carcinoma. BMC Cancer. 2018;18(1):1131.

16. Liu C, Xing W, Si T, Yu H, Guo Z. Efficacy and safety of apatinib combined with transarterial chemoembolization for hepatocellular carcinoma with portal venous tumor thrombus: a retrospective study. Oncotarget. 2017;8(59):100734-45.

17. Kudo M, Han KH, Ye SL, Zhou J, Huang YH, Lin SM, et al. A Changing Paradigm for the Treatment of Intermediate-Stage Hepatocellular Carcinoma: Asia-Pacific Primary Liver Cancer Expert Consensus Statements. Liver Cancer. 2020;9(3):245-60.

18. Shim SJ, Seong J, Han KH, Chon CY, Suh CO, Lee JT. Local radiotherapy as a complement to incomplete transcatheter arterial chemoembolization in locally advanced hepatocellular carcinoma. Liver Int. 2005;25(6):1189-96.

19. Lencioni R. Loco-regional treatment of hepatocellular carcinoma. Hepatology. 2010;52(2):762-73.

20. Iwai Y, Terawaki S, Honjo T. PD-1 blockade inhibits hematogenous spread of poorly immunogenic tumor cells by enhanced recruitment of effector T cells. Int Immunol. 2005;17(2):133-44.

21. Lee MS, Ryoo BY, Hsu CH, Numata K, Stein S, Verret W, et al. Atezolizumab with or without bevacizumab in unresectable hepatocellular carcinoma (GO30140): an open-label, multicentre, phase 1b study. Lancet Oncol. 2020;21(6):808-20.

22. Rahma OE, Hodi FS. The Intersection between Tumor Angiogenesis and Immune Suppression. Clin Cancer Res. 2019;25(18):5449-57.

23. Griffioen AW, Damen CA, Martinotti S, Blijham GH, Groenewegen G. Endothelial intercellular adhesion molecule-1 expression is suppressed in human malignancies: the role of angiogenic factors. Cancer Res. 1996;56(5):1111-17.

24. Osada T, Chong G, Tansik R, Hong T, Spector N, Kumar R, et al. The effect of anti-VEGF therapy on immature myeloid cell and dendritic cells in cancer patients. Cancer Immunol Immunother. 2008;57(8):1115-24.

25. Terme M, Colussi O, Marcheteau E, Tanchot C, Tartour E, Taieb J. Modulation of immunity by antiangiogenic molecules in cancer. Clin Dev Immunol. 2012;2012:492920.

26. Kusmartsev S, Eruslanov E, Kübler H, Tseng T, Sakai Y, Su Z, et al. Oxidative stress regulates expression of VEGFR1 in myeloid cells: link to tumor-induced immune suppression in renal cell carcinoma. J Immunol. 2008;181(1):346-53.

27. Terme M, Pernot S, Marcheteau E, Sandoval F, Benhamouda N, Colussi O, et al. VEGFA-VEGFR pathway blockade inhibits tumor-induced regulatory T-cell proliferation in colorectal cancer. Cancer Res. 2013;73(2):539-49.

28. Finn RS, Qin S, Ikeda M, Galle PR, Ducreux M, Kim TY, et al. Atezolizumab plus Bevacizumab in Unresectable Hepatocellular Carcinoma. N Engl J Med. 2020;382(20):1894-905.

29. Lu W, Jin XL, Yang C, Du P, Jiang FQ, Ma JP, et al. Comparison of efficacy between TACE combined with apatinib and TACE alone in the treatment of intermediate and advanced hepatocellular carcinoma: A single-center randomized controlled trial. Cancer Biol Ther. 2017;18(6):433-8.

30. Semenza GL. Targeting HIF-1 for cancer therapy. Nat Rev Cancer. 2003;3(10):721-32.

31. Schicho A, Hellerbrand C, Krüger K, Beyer LP, Wohlgemuth W, Niessen C, et al. Impact of Different Embolic Agents for Transarterial Chemoembolization (TACE) Procedures on Systemic Vascular Endothelial Growth Factor (VEGF) Levels. J Clin Transl Hepatol. 2016;4(4):288-92.

32. Carmeliet P. Angiogenesis in life, disease and medicine. Nature. 2005;438(7070):932-6.

33. Poon RT, Lau C, Yu WC, Fan ST, Wong J. High serum levels of vascular endothelial growth factor predict poor response to transarterial chemoembolization in hepatocellular carcinoma: a prospective study. Oncol Rep. 2004;11(5):1077-84.

34. Chao Y, Li CP, Chau GY, Chen CP, King KL, Lui WY, et al. Prognostic significance of vascular endothelial growth factor, basic fibroblast growth factor, and angiogenin in patients with resectable hepatocellular carcinoma after surgery. Ann Surg Oncol. 2003;10(4):355-62.

35. Britten CD, Gomes AS, Wainberg ZA, Elashoff D, Amado R, Xin Y, et al. Transarterial chemoembolization plus or minus intravenous bevacizumab in the treatment of hepatocellular cancer: a pilot study. BMC Cancer. 2012;12:16.

36. Buijs M, Reyes DK, Pawlik TM, Blackford AL, Salem R, Messersmith WA, et al. Phase 2 trial of concurrent bevacizumab and transhepatic arterial chemoembolization in patients with unresectable hepatocellular carcinoma. Cancer. 2013;119(5):1042-9.

37. Pinter M, Ulbrich G, Sieghart W, Kölblinger C, Reiberger T, Li S, et al. Hepatocellular Carcinoma: A Phase II Randomized Controlled Double-Blind Trial of Transarterial Chemoembolization in Combination with Biweekly Intravenous Administration of Bevacizumab or a Placebo. Radiology. 2015;277(3):903-12.

38. Pinato DJ, Murray SM, Forner A, Kaneko T, Fessas P, Toniutto P, et al. Trans-arterial chemoembolization as a loco-regional inducer of immunogenic cell death in hepatocellular carcinoma: implications for immunotherapy. J Immunother Cancer. 2021;9(9).

39. Wissniowski TT, Hänsler J, Neureiter D, Frieser M, Schaber S, Esslinger B, et al. Activation of tumor-specific T lymphocytes by radio-frequency ablation of the VX2 hepatoma in rabbits. Cancer Res. 2003;63(19):6496-500.

40. Dromi SA, Walsh MP, Herby S, Traughber B, Xie J, Sharma KV, et al. Radiofrequency ablation induces antigen-presenting cell infiltration and amplification of weak tumor-induced immunity. Radiology. 2009;251(1):58-66.

41. Cui J, Wang N, Zhao H, Jin H, Wang G, Niu C, et al. Combination of radiofrequency ablation and sequential cellular immunotherapy improves progression-free survival for patients with hepatocellular carcinoma. Int J Cancer. 2014;134(2):342-51.

42. Wang X, Liu G, Chen S, Bi H, Xia F, Feng K, et al. Combination therapy with PD-1 blockade and radiofrequency ablation for recurrent hepatocellular carcinoma: a propensity score matching analysis. Int J Hyperthermia. 2021;38(1):1519-28.

43. Duffy AG, Ulahannan SV, Makorova-Rusher O, Rahma O, Wedemeyer H, Pratt D, et al. Tremelimumab in combination with ablation in patients with advanced hepatocellular carcinoma. J Hepatol. 2017;66(3):545-51.

44. McDermott DF, Huseni MA, Atkins MB, Motzer RJ, Rini BI, Escudier B, et al. Clinical activity and molecular correlates of response to atezolizumab alone or in combination with bevacizumab versus sunitinib in renal cell carcinoma. Nat Med. 2018;24(6):749-57.

45. Pinato DJ, Guerra N, Fessas P, Murphy R, Mineo T, Mauri FA, et al. Immune-based therapies for hepatocellular carcinoma. Oncogene. 2020;39(18):3620-37.
